# *Klebsiella* phages representing a novel clade of viruses with an unknown DNA modification and biotechnologically interesting enzymes

**DOI:** 10.1007/s00253-016-7928-3

**Published:** 2016-10-21

**Authors:** Barbara Maciejewska, Bartosz Roszniowski, Akbar Espaillat, Agata Kęsik-Szeloch, Grazyna Majkowska-Skrobek, Andrew M. Kropinski, Yves Briers, Felipe Cava, Rob Lavigne, Zuzanna Drulis-Kawa

**Affiliations:** 1 Institute of Genetics and Microbiology, University of Wroclaw, S. Przybyszewskiego 63/77, 51-148 Wrocław, Poland; 2 Laboratory for Molecular Infection Medicine Sweden, Molecular Biology Department, Umeå University, SE-901 87 Umeå, Sweden; 3 Departments of Food Science, Molecular and Cellular Biology, and Pathobiology, University of Guelph, 50 Stone Road East, Guelph, ON N1G 2W1 Canada; 4 Department Applied Biosciences, Ghent University, Valentin Vaerwyckweg 1, 9000 Ghent, Belgium; 5 Laboratory of Gene Technology, KULeuven, Leuven Kasteelpark Arenberg 21 Box 2462, 3001 Leuven, Belgium

**Keywords:** Bacteriophage, *Klebsiella* spp., Kp15virus, Thermostable endolysin, DNA modification

## Abstract

Lytic bacteriophages and phage-encoded endolysins (peptidoglycan hydrolases) provide a source for the development of novel antimicrobial strategies. In the present study, we focus on the closely related (96 % DNA sequence identity) environmental myoviruses vB_KpnM_KP15 (KP15) and vB_KpnM_KP27 (KP27) infecting multidrug-resistant *Klebsiella pneumoniae* and *Klebsiella oxytoca* strains. Their genome organisation and evolutionary relationship are compared to *Enterobacter* phage phiEap-3 and *Klebsiella* phages Matisse and Miro. Due to the shared and distinct evolutionary history of these phages, we propose to create a new phage genus “Kp15virus” within the *Tevenvirinae* subfamily. In silico genome analysis reveals two unique putative homing endonucleases of KP27 phage, probably involved in unrevealed mechanism of DNA modification and resistance to restriction digestion, resulting in a broader host spectrum. Additionally, we identified in KP15 and KP27 a complete set of lysis genes, containing holin, antiholin, spanin and endolysin. By turbidimetric assays on permeabilized Gram-negative strains, we verified the ability of the KP27 endolysin to destroy the bacterial peptidoglycan. We confirmed high stability, absence of toxicity on a human epithelial cell line and the enzymatic specificity of endolysin, which was found to possess endopeptidase activity, cleaving the peptide stem between l-alanine and d-glutamic acid.

**Electronic supplementary material:**

The online version of this article (doi:10.1007/s00253-016-7928-3) contains supplementary material, which is available to authorized users.

## Introduction

Lytic bacteriophages (viruses that attack and lyse bacteria) are ubiquitous in nature. As natural predators, they control the bacterial population and have a large impact on bacterial ecosystems (Weinbauer and Rassoulzadegan 2004). The interest in phages has recently increased due to their antibacterial potential (including lytic enzymes—endolysins). Particular attention has been drawn to phages and endolysins which demonstrate activity on highly virulent and multidrug-resistant pathogens including *Klebsiella pneumoniae*. This bacterium is associated with nosocomial infections but also commonly found in natural environments, such as water reservoirs, soil, sewage and on plant surface (Bagley 1985). Moreover, environmental strains of *K. pneumoniae* have been shown to be equally virulent as clinical isolates (Podschun et al. 2001; Struve and Krogfelt 2003). Particularly noteworthy is the occurrence of different *Klebsiella* spp. capable of expressing virulence factors including serum resistance, capsular polysaccharides, pili and siderophores (Podschun et al. 2001). The spread of highly virulent and antibiotic-resistant *K. pneumoniae* strains, both in hospitals and in natural environment, also requires a more in-depth knowledge about *Klebsiella*-specific bacteriophages, as natural enemies of these bacteria and as a potential source of antimicrobials to control *Klebsiella* infections. To date, 28 genomes of dsDNA phages specific to *Klebsiella* have been deposited into the NCBI database. These have been classified within three families: Podoviridae (14), Myoviridae (9) and Siphoviridae (5). The present study focusses on the genome organisation of two myoviruses, vB_KpnM_KP15 (KP15) and vB_KpnM_KP27 (KP27), and adding the in silico characterisation of their proteins (including division to the protein function groups), as well as complete annotation of their regulatory sequences to the previously submitted GenBank files (GU295964 and HQ918180). Furthermore, the detailed comparative genomic analysis was performed including recently described Miro and Matisse phages, as well as phiEap-3, similar to KP15 and KP27. The phages were isolated from environmental sewage samples in Poland and propagated on extended-spectrum beta-lactamases (ESBL) producing, multidrug-resistant *K. pneumoniae* strains. A basic microbiological description of these phages was described previously (Kęsik-Szeloch et al. 2013). In this paper, detailed genomic analysis of both phages is reported and placed into their taxonomic context. Special attention has been placed on the detection and description of genes essential from the application perspective to control the bacterial population: (i) encoding tail fibres and determining host specificity, (ii) responsible for DNA modification and thereby DNA resistance to host enzyme digestion, and (iii) encoding proteins involved in host lysis including experimental study of endolysin activity.

## Materials and methods

### Propagation and purification of phages

*K. pneumoniae*-specific bacteriophages were isolated from sewage samples by enrichment technique described previously (Drulis-Kawa et al. 2011; Kęsik-Szeloch et al. 2013). Strain *K. pneumoniae* ATCC 700603 and clinical strain *K. pneumoniae* 767 ESBL(+) (Polish Collection of Microorganisms (PCM) B/F/00064) were used for amplification of KP15 (PCM F/00063) and KP27 (PCM F/00064) respectively. The bacteriophage titer in the supernatant was determined using the double-agar layer technique according to Adams (1959). Reisolation of single plaques was performed to obtain a pure phage isolates. Phage DNA was isolated according to the modified protocol for λ DNA isolation (Ceyssens et al. 2009).

### Genome analysis

The vB_KpnM_KP15 and vB_KpnM_KP27 genomes were sequenced commercially (Genomed Ltd. Warsaw, Poland). The annotated sequences of phage DNA have been deposited in GenBank under accession numbers GU295964 and HQ918180 for KP15 and KP27 respectively. During the comparison process, CLUSTAL Omega (Sievers et al. 2011), NCBI BLASTN (Altschul et al. 1990) and BLASTP (Gish and States 1993) tools were used to verify the level of identity among genes encoding homologous proteins. The obtained data was grouped in Table S1 (Online Resource 1) and used for building input files for visualisation by Circos (Krzywinski et al. 2009). The search for conserved motifs including potential promoters was performed using MEME/MAST (Bailey et al. 2009) on 100 nt sequences, directly upstream of each open reading frame (ORF). Putative Rho-independent terminators were marked with the ARNold software (Gautheret and Lambert 2001), whereas the search of the transfer RNA (tRNA) was performed using ARAGORN (Laslett and Canbäck 2004) and confirmed by tRNAscan-SE v. 2.0 (Lowe and Chan 2016). To search for gene products involved in DNA modification, we specifically looked for gene products as described by Iyer et al. (2013). Iterative sequence profile searches were performed using the PSI-BLAST (http://www.ncbi.nlm.nih.gov/blast/) and HMMER (Eddy 1998). The proteins involved in lysis were identified by comparative proteomic analysis based on amino acid sequence similarity (BLAST). Conserved domains were recognized by NCBI’s BLAST, HMMER and Phyre-2 (Kelley et al. 2015). To detect of transmembrane domains, TMHMM server 2.0 (Krogh et al. 2001) was used. Signal peptides were predicted in SignalP 4.1 (Petersen et al. 2011). Physicochemical properties of analysed proteins were established using ProtParam (ExPASy) (Gasteiger et al. 2005), and protein binding regions have been predicted with PredictProtein server (Rost et al. 2004). The genomes were initially annotated using AutoFACT (Koski et al. 2005) and subsequently verified manually using Kodon (Applied Maths, Austin, TX, USA). We screened for ORFs with a length ≥75 nt preceded by a visually recognizable ribosome-binding site.

### Recombinant endolysin preparation

The recombinant endolysin was prepared according to methods described previously by Walmagh et al. (2013). Briefly, the coding sequence for the KP27 endopeptidase was amplified from genomic DNA by PCR using KAPA HiFi DNA Polymerase (Kapa Biosystems, Wilmington, MA, USA) and ORF-specific primers. The amplified endolysin gene was cloned into the commercially available pEXP-5-CT/TOPO® TA Expression vector (Invitrogen, Thermo Fisher Scientific, Waltham, MA, USA) according to the manufacturer’s recommendations, and BL21 (DE3) pLysS (Agilent Technologies, Santa Clara, CA, USA) was transformed with the expression construct. Transformants containing a Sanger sequence-verified construct were cultured at 37 °C with shaking (200 rpm) to achieve of mid-exponential-phase (OD_600_ ∼0.5–0.7). Next, expression was induced by the addition of isopropyl-β-d-thiogalactopyranoside (IPTG, final concentration of 0.5 mM), and bacteria further culture additional 18 h at 20 °C. After expression, cells were collected by centrifugation, suspended in lysis buffer, and the cell wall was disrupted by a combination of three times freeze–thawing and sonication. The recombinant protein was purified from the filtered supernatant by affinity chromatography using NGC Medium Pressure Chromatography Systems (Bio-Rad, Hercules, CA, USA) combined with 5-ml nickel columns: Bio-Scale Mini Profinity IMAC Cartridges (Bio-Rad, Hercules, CA, USA). The fractions containing recombinant protein were eluted by elution buffer containing 500 mM imidazole and pooled. Protein was dialyzed against phosphate-buffered saline pH 7.4 and analysed by SDS-PAGE (Fig. S1, Online Resource 1). The concentration of purified recombinant enzyme was then determined fluorimetrically (Qubit® Protein Assay Kit, Molecular Probes, Thermo Fischer Scientific, Waltham, MA, USA).

### Determination of peptidoglycan degrading activity of KP27 endolysin

The peptidoglycan degrading activity of the purified endolysin was determined on outer membrane permeabilized strains: *K. pneumoniae* ATCC 700603, *K. pneumoniae* clinical isolate 486 (PCM B/F/00068), *Pseudomonas aeruginosa* PAO1 (ATCC 15692), *Salmonella enterica* subsp. *enterica* serotype Typhimurium LT2 (ATCC 700720) and *Escherichia coli* ATCC 25922. The strains were permeabilized by resuspension of the cells in a chloroform-saturated Tris–HCl buffer according to Lavigne et al. (2004). The small amounts of chloroform dissolve the outer and inner membrane but leave the peptidoglycan saccule intact. Each strain was grown until mid-exponentially growth phase (OD_600_ of 0.6) and then the cells were collected by centrifugation and incubated for 45 min in a chloroform-saturated 0.05 M Tris–HCl buffer (pH 7.7). Subsequently, cells were washed in PBS (pH 7.4) and concentrated to an OD_600_ of 1.0. The peptidoglycan degrading activity was determined according to Briers et al. (2007) and relies on the spectrophotometric measurement of drop in turbidity of the outer membrane permeabilized cell suspension in the presence of endolysin. The peptidoglycan degrading activity corresponding to one unit equal the amount of enzyme causing an OD_600_ decrease of 0.001/min (Briers et al. 2007).

### Peptidoglycan isolation and analysis

A 200-ml overnight stationary phase culture of *E. coli* was pelleted at 4500×*g* and resuspended in 5 ml of PBS and then added to an equal volume of 10 % SDS in a boiling water bath and vigorously stirred for 4 h, then stirred overnight at RT. The insoluble fraction (peptidoglycan) was pelleted at 400,000×*g* for 15 min at 30 °C (TLA-100.3 rotor; OptimaTM Max Ultracentrifuge, Beckman Coulter, Brea, CA, USA). After the SDS was washed out, peptidoglycan was treated with Pronase E 0.1 mg/ml at 60 °C for 1 h and further boiled in 1 % SDS for 2 h to stop the reaction. After SDS was removed as described previously, sacculi were resuspended in 200 μl of 50 mM sodium phosphate buffer pH 4.9 and digested overnight with 30 μg/ml muramidase (Cellosyl, Hoechst, Frankfurt, Germany) at 37 °C. Muramidase digestion was stopped by incubation in a boiling water bath (5 min). Coagulated protein was removed by centrifugation. The supernatants were mixed with 150 μl 0.5 M sodium borate pH 9.5 and subjected to reduction of muramic acid residues into muramitol by sodium borohydride treatment (10 mg/ml final concentration, 30 min at room temperature). Samples was adjusted to pH 3.5 with phosphoric acid. Chromatographic analyses of muropeptides have been performed on an ACQUITY Ultra Performance Liquid Chromatography (UPLC) BEH C18 column (130 Å, 1.7 μm, 2.1 mm × 150 mm; Waters, Milford, MA, USA), and peptides were detected at Abs. 204 nm using an ACQUITY UPLC UV–Vis Detector. Muropeptides were separated using a linear gradient from buffer A (phosphate buffer 50 mM, pH 4.35) to buffer B (phosphate buffer 50 mM, pH 4.95 methanol 15 % (*v*/*v*) in 20 min, and flow 0.25 ml/min). For formulation, muropeptides were analysed with Agilent 6550 iFunnel Q-TOF LC/MS System (Agilent Technologies, Santa Clara, CA, USA), with the same gradient.

### In vitro peptidoglycan digestion

Peptidoglycan digestions were performed on 90 min reactions for KP27 endolysin and muramidase. Stationary phase of *E. coli* sacculi (0.4 mg/ml) was incubated with 30 μg/ml muramidase or 0.4 mg/ml of KP27 on a Tris–HCl 20 mM pH 8, 1 mM MgCl_2_ and 1 mM ZnCl_2_. Prior to the reaction with a second enzyme, the individual enzymatic reaction was heat inactivated for 10 min at 100 °C.

### Endolysin stability assays

The stability of endolysin was determined in PBS buffer (pH 7.4) by monitoring the peptidoglycan degrading activity after 1-month storage at 4 °C. The thermostability was determined in PBS buffer (pH 7.4) after 15 and 60 min incubation at 50, 60, 70, 80, 90 and 110 °C. The pH stability was assessed after 1 h incubation in citric acid–Na_2_HPO_4_ buffer solution (pH 2.6, pH 4, pH 5) and in sodium carbonate–sodium bicarbonate buffer solution (pH 10).

### Evaluation of endolysin cytotoxicity

The cytotoxic effect of KP27 endolysin was evaluated using the human lung carcinoma epithelial cell line A549 (CCL-185, ATCC), based on three different assays. Briefly, 24 h before addition of endolysin, the exponentially growing cells (from 1 × 10^4^ to 2.5 × 10^4^) were plated in flat-bottom 96-well microplates (Nunc, Thermo Fischer Scientific, Waltham, MA, USA) and incubated in 100 μl of complete growth medium containing DMEM (Lonza, Basel, Switzerland) supplemented with 2 mM/l glutamax, 1 % antibiotic–antimycotic solution and 10 % heat-inactivated fetal bovine serum (all from Gibco BRL, Thermo Fischer Scientific, Waltham, MA, USA). After 24 h of incubation, aliquots of 100 μl of various concentrations of endolysin (from 1 to 50 μg/ml) were added, and the cells were further incubated for 24 and 48 h. The cell cultures were maintained at 37 °C in a humidified atmosphere of 5 % CO_2_/95 % air. First, to determine the number of viable cells through measurement of their reducing potential, the MTT colorimetric assay was performed as described previously (Mosmann 1983). The concentration of formazan was determined by an optical density measurement at 570 nm using the ASYS UVM 340 (Biochrom Ltd., Cambourne, UK) spectrophotometer. As an alternative method to quantitate cell viability, the trypan blue dye exclusion method was used. Endolysin-treated cells were assayed by adding trypan blue solution (0.4 % in PBS; Sigma-Aldrich, Poznan, Poland) to the culture medium. After 3 min, the number of dead cells that retained the dye was compared to the total number of cells to calculate cell viability. Third, to measure changes in membrane integrity that occur as a result of cell death, the CellTox Green Cytotoxicity Assay (Promega, Mannheim, Germany) was performed according to the manufacturer’s instruction. Following addition of a single reagent directly to cells cultured in serum-supplemented medium, the fluorescence was determined using GloMax Discover Multimode Plate Reader (Promega) following 15 min of incubation, as a positive control 3-bromopyruvate (Sigma-Aldrich, Poland) was used.

## Results

### Genome properties

Two closely related environmental bacteriophages vB_KpnM_KP15 and vB_KpnM_KP27 with genomes of 174,436 and 174,413 bp, lytic to *K. pneumoniae*, show a high level of DNA identity (96 %, based on BLASTN comparison). As an addition to existing annotation, the regulatory sequence positions were localized using MEME/MAST (promoters) and ARNold (terminators) software. Despite this high level of identity, the genomes differ significantly: 53 and 118 rho-independent terminators have been predicted for phage KP15 and KP27 respectively (Tables S2 and S3, Online Resource 1). Seventeen promoters in phage KP15 genome and 41 in phage KP27 were localized. These differences can be explained by the following: (i) the level of point mutations in intragenic regions of KP15 is probably high, which may influence the formation of −10 and −35 boxes leading to promoter inactivation; (ii) the viral promoters with a high variety of motif structure are present in KP15 genome, preventing the determination of their position via current in silico analysis tools; (iii) phage KP15 needs less number of promoters for sufficient transcription or uses the host regulatory sequences. The search for tRNA sequences using ARAGORN, revealed one tRNA-Met with CAT anticodon in both viral genomes, while tRNAscan-SE v. 2.0 confirmed ARAGORN result and revealed second tRNA in both phages—tRNA-Gln with CTG anticodon.

### Comparative genome analysis

**Fig. 1:**
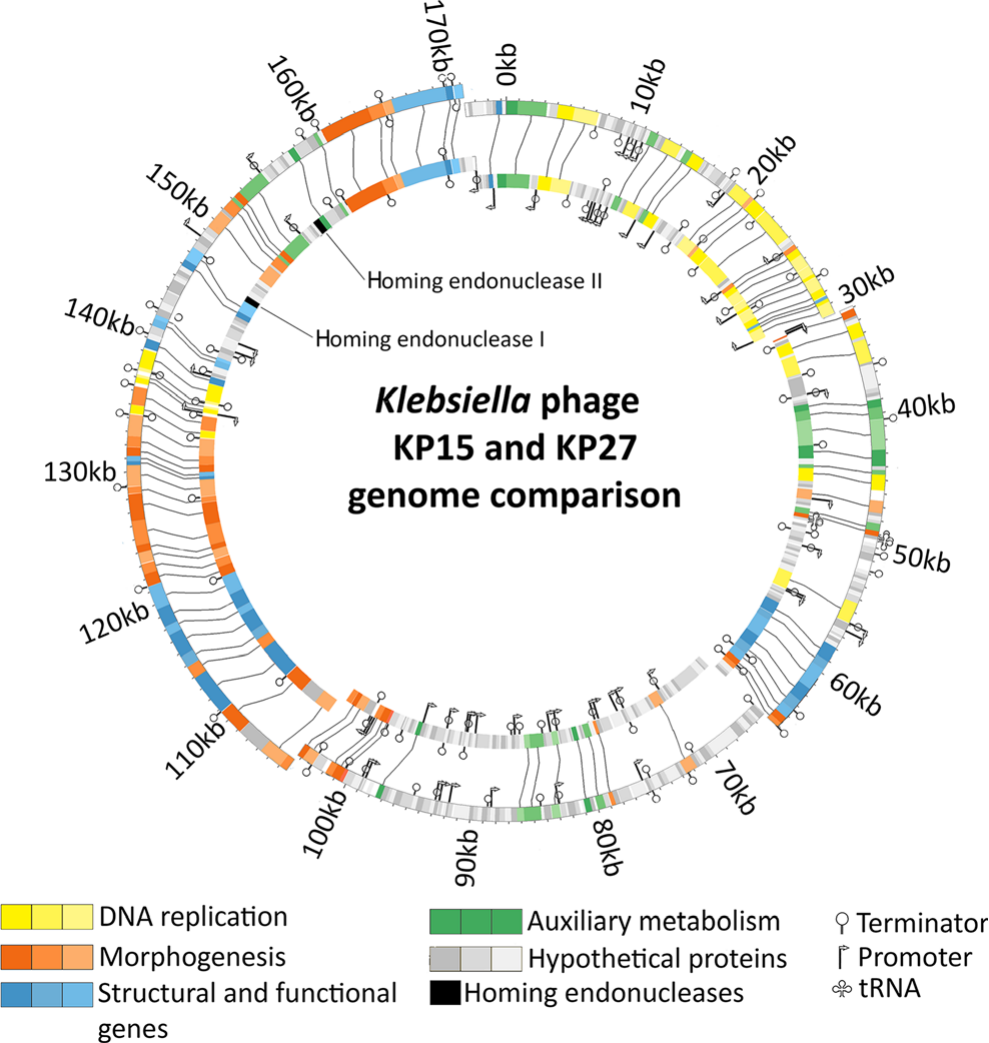
The graphical comparison of KP15 and KP27 genomes. The *outer ring* of the ideogram represents phage KP15 genome, while the *inner ring* shows phage KP27 genome. Differences were marked inside the *inner circle*, indicating proper name for particular genes. *Lines* that connect two rings are linking proteins that are homologues or fulfil similar function for both phages. Genes have been grouped according to their predicted function: DNA replication, morphogenesis genes, auxiliary metabolism, structural and additional genes with known function, as well as homing endonuclease genes, present only in KP27 genome (coloured black)

**Fig. 2:**
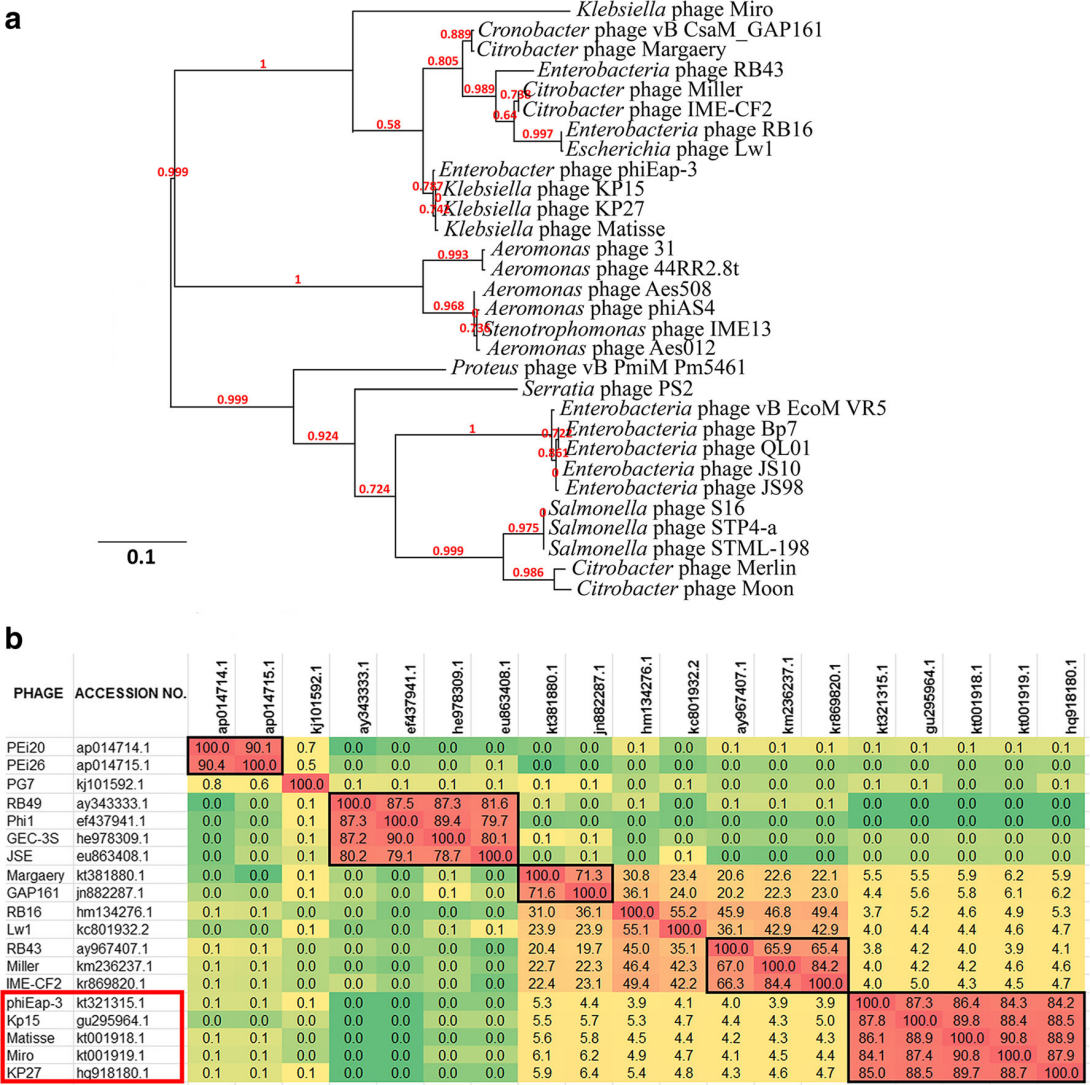
The similarities of “Kp15virus” and related species of bacteriophages. **a** Phylogenetic analysis based on large subunit terminase proteins. **b** Phylogenomic tree calculated using Gegenees 2.2.1 based on pairwise comparisons

The graphical comparison of both genomes is presented in a Circos figure (Fig. 1). The early genes can be linked to the auxiliary metabolism and the conversion to the phage infection metabolism, followed by proteins involved in DNA replication (with the exception of the small region of additional predicted auxiliary metabolism genes). Middle genes are the two smaller regions of the structural and additional functional genes, flanking the significant region of hypothetical genes. Morphogenesis-associated proteins are localized in the remaining part of the genome (late genes), although the sequences which represent auxiliary metabolism, DNA replication and morphogenesis genes are also occasionally present. The full list of KP15 and KP27 annotated genes, including those genes unique to phage KP27, have been listed in Supplementary Table S1 (Online Resource 1), which also contains the input data needed for the generation of Circos genome maps. In addition, the genomes of KP15 and KP27 phages have been compared to three other phages, specifically *Enterobacter* phage phiEap-3 (accession no. KT321315); *Klebsiella* phage Matisse (KT001918) and *Klebsiella* phage Miro (KT001919) with the same genome organisation and 95 % identity to KP15 and KP27 (based on BLASTN) (Fig. S2, Online Resource 1). Moreover, the phylogenetic analysis of the large subunit terminase proteins of KP15-like viruses and variety of other similar phages constructed using “one click” was made using phylogeny.fr (Dereeper et al. 2008). The Hoody T-like capsid gene contained a frameshift which was corrected prior to this analysis. By default, the pipeline was set up to run and connect programs recognized for their accuracy and speed (MUSCLE for multiple alignment and PhyML for phylogeny) to reconstruct a robust phylogenetic tree from a set of sequences. It also included the use of Gblocks to eliminate poorly aligned positions and divergent regions (Fig. 2a). Phylogenomic tree of “Kp15virus” and related species of bacteriophages was also calculated using Gegenees 2.2.1 based on pairwise comparisons of the analysed sequences (BLASTN method with “custom” settings of fragmenting algorithm—size 100 bp, shift 50 bp) (Fig. 2b). Phage Miro also shares 96.1 % common proteins with phage KP15 as shown using CoreGenes 3.5. and progressive Mauve analysis reveals that these genomes are related and collinear (data presented in *Taxonomy Proposal*). Although the Miro phage was more distantly related to the other four based on the high identity of the large subunit terminase, the overall high similarity of the genomes allows to propose creation of a new genus Kp15virus, within the *Tevenvirinae* subfamily, named after group type species KP15 (https://talk.ictvonline.org/files/proposals/taxonomy_proposals_prokaryote1/m/bact02/6304).

### Do homing endonucleases regulate phage DNA resistance towards host restriction endonucleases and, as a consequence, impact host range?

The two interesting genes found in KP27 genome (Fig. 1), located in positions 146,039–146,557 (173 aa) and 155,517–156,158 (213 aa), were recognized as putative homing endonuclease genes (YP_007348875 and YP_007348891). Such, HNH endonuclease homologues are widespread among *Caudovirales* and usually interrupt DNA metabolism genes as well as late genes.

**Fig. 3:**
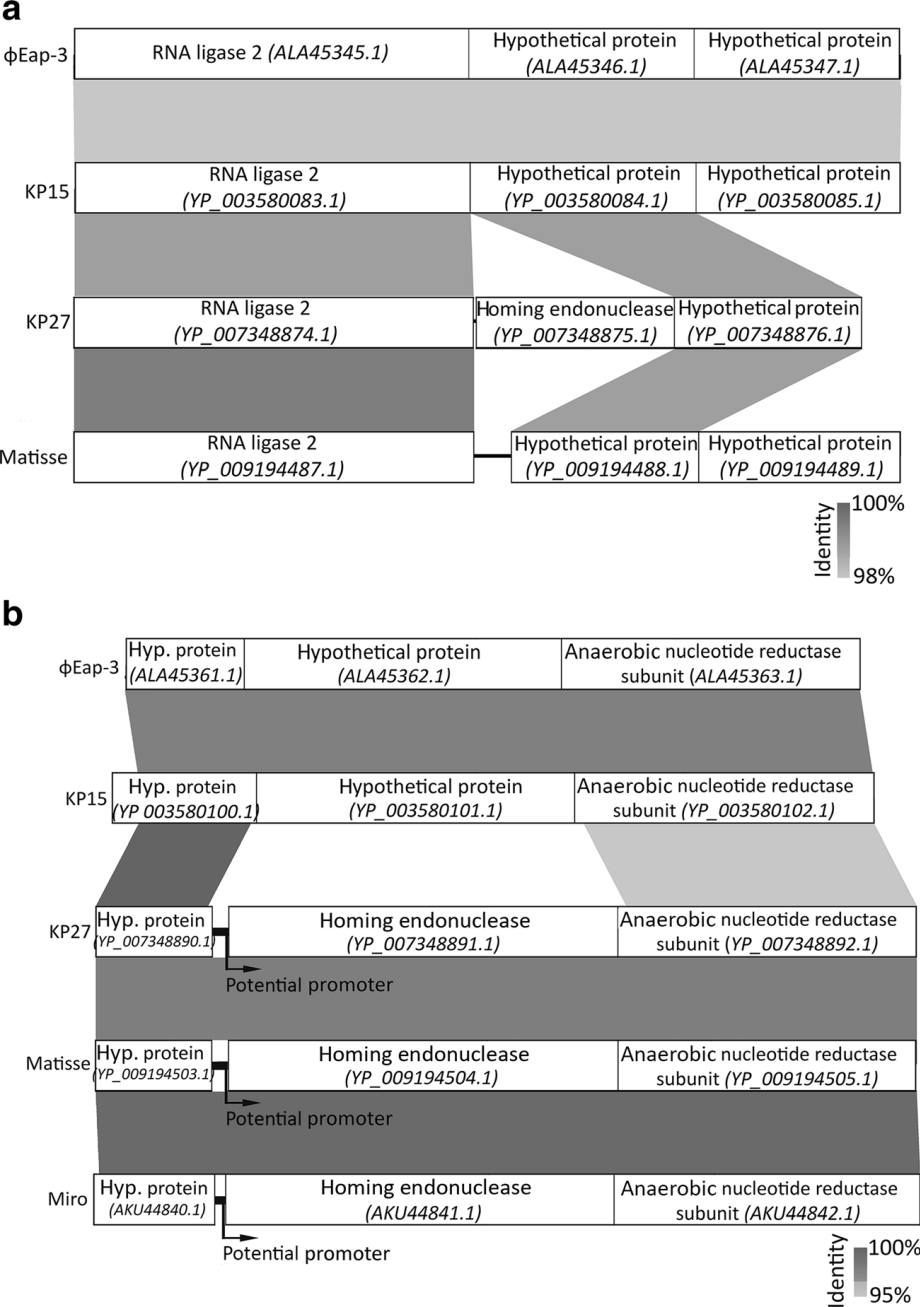
The similarity of YP_007348875 (**a**) and YP_007348891 (**b**) homing endonucleases region among the “Kp15virus” representatives

As homing endonucleases are expected to splice genes and introduce themselves as the introns, length and completion of the genes lying directly next to the homing endonucleases were reanalysed using BLASTP (Fig. 3). The identification of homologous homing endonuclease YP_007348875 region was possible in all “Kp15 virus” phages, except in Miro (Fig. 3a). The KP27 HNH endonuclease is placed between RNA ligase 2 and a hypothetical protein. The hypothetical protein that lies downstream has a BLASTP identity of 100 % (e = 3e^−126^ − 2e^−123^) to the proteins that lie directly after RNA ligase 2 in compared phages. In the case of the second KP27 homing endonuclease (YP_007348891), the homologous region was found in all five compared phages (Fig. 3b). Both proteins (hypothetical protein and anaerobic nucleotide reductase subunit) lying directly next to homing endonuclease hold high homology in each phage. Moreover, upstream of the YP_007348891, a region that may be considered as the promotor regulating the transcription of the downstream genes (including the anaerobic nucleotide reductase subunit), has been found. The similar regulatory sequences located in homologous regions were not found neither in phage KP15 nor in phiEap-3 but were located in Miro and Matisse phages, with BLASTP identity of 100 % (e = 0.0).

A detailed analysis to identify possible genes involved in DNA modification was performed, which revealed a predicted Dcm and Dam methylase. However, homologues for these enzymes were found in all Kp15virus members, which do not correlate to the restriction resistance observed (Table 1). In addition, other methyltransferases, hydroxymethyltransferases, glycosyltransferases, TET/JBP proteins, Mom enzymes or proteins involved in synthesis of glutamylthymine, putrescinylthymine, deazapurines and *S*-adenosylmethionine (SAM) derivatives (Iyer et al. 2013) were not found (Table S4, Online Resource 1). These elements reinforce the hypothesis that this phage is using a HNH-endonuclease-mediated regulation of DNA modification. In turn, this potent resistance of KP27 phage DNA to restriction digestion can be associated to a broader lytic spectrum compared to KP15, based on phage susceptibility tested on 222 *Klebsiella* spp. strains. KP27 propagates on 22 % of *K. pneumoniae* and 37 % of *K. oxytoca* isolates, compared to 9 and 35 % for KP15 respectively (Kęsik-Szeloch et al. 2013). Indeed, the strong similarity (>95 %) of the tail fibre elements (except L-shaped tail fibre), as shown in Fig. S3 (Online Resource 1), does not explain the differences in host range. As expected, no similarities of the middle part of L-shaped tail fibre were noticed, which may condition differences in enzymatic domain content responsible for specific host polysaccharide cleavage activity. Nevertheless, the C-terminal end of L-shaped tail fibre, which is considered to be responsible for host receptors recognition (Garcia-Doval and van Raaij 2012), is highly conserved for KP15 and KP27.

**Table 1:** The presence of DNA modifying genes among “Kp15virus” representatives

Phage	Endonuclease I	Endonuclease II	Dcm methylase	Dam methylase
KP27	Origin of YP_007348875.1 Location: 146,039–146,557 gi: 448,260,781	Origin of YP_007348891.1 Location: 155,517–156,158 gi: 448,260,797	Origin of YP_007348760.1 Location: 72,061–71,150 gi: 448,260,797	Origin of YP_007348882.1 Location: 150,190–151,041 gi: 448,260,788
KP15	Not present	Not present	Similarity: 99 % Location: 72,652–71,726 gi: 294,661,528	Similarity: 98 % Location: 151,063–151,914 gi: 294,661,636
φEap-3	Not present	Not present	Similarity: 99 % Location: 47,170–47,676 gi: 921,955,723	Similarity: 98 % Location: 151,388–152,239 gi: 921,955,896
Matisse	Not present	Similarity: 98 % Location: 158,336–158,977 gi: 910,308,875	Similarity: 98 % Location: 48,820–49,326 gi: 910,308,692	Similarity: 98 % Location: 152,683–153,517 gi: 910,308,865
Miro	Not present	Similarity: 98 % Location: 158,538–159,179 gi: 910,309,153	Similarity: 94 % Location: 73,331–72,390 gi: 910,309,019	Similarity: 82 % Location: 153,016–153,741 gi: 910,309,143

### Description of KP15 and KP27 lysis system

KP15 and KP27 phages possess the most advanced lysis system among lytic phages infecting Gram-negative hosts, composed of four proteins: a holin, antiholin, spanin and endolysin. These proteins are highly similar (>99 % similarity) between both phages. Similarly to *Enterobacteria* phage T4, the KP15 and KP27 lysis genes are spread throughout the phage genome. Holins are proteins able to form a nonspecific membrane lesion, essential for the transfer of endolysins from the cytoplasm to the periplasmic space (Young and Bläsi 1995; Young 2002). Those of KP15 and KP27 (ADE35068 and AEX26746 respectively) are relatively large in size (215 aa; 24 kDa) with one transmembrane domain which classifies analysed proteins as class III holins. The observed large size of holins is explained by its predicted 168 residue-long, C-terminal periplasmic domain, characteristic of the holin T superfamily group (Ramanculov and Young 2001; Moussa et al. 2012). The probable role of C-terminal part of antiholin is binding to C-terminal domain of holin and producing an inactive holin–antiholin dimer and thereby lysis retardation (Tran et al. 2005). In KP15 and KP27 phages, holin activity is predicted to be regulated by the antiholin ADE34945 and AEX26616 respectively. Additionally, both phages encode a bimolecular spanin complex predicted to be responsible for outer membrane destabilisation and release of progeny virions at the end of lytic cycle (Young 2014). The KP15 and KP27 spanin genes are located side by side in both genomes, where the predicted o-spanins of KP15 and KP27 (ADE34895 and AEX26552 respectively) are entirely embedded within the genes encoding the i-spanins (ADE34894 and AEX26551) in the +1 reading frame. The predicted endolysins of both phages responsible for peptidoglycan degradation (ADE34958 and AEX26632) differ only in one amino acid (glutamic acid of KP15 endolysin is replaced by glutamine in the KP27 endolysin in the seventeenth position from N-terminus). The endolysin is a relatively small protein (131 residues, 14.7 kDa) of globular structure, possessing a single, enzymatically active conserved domain, which based on in silico analysis, belongs to VanY (pfam 02557 superfamily, part of the peptidase M15 family). VanY is predicted to show d-alanyl-d-alanine carboxypeptidase cleaving the terminal d-Ala residue from the stem peptide with affecting the peptidoglycan meshwork integrity.

### Experimental analysis of the endolysin reveals interesting properties towards biotechnological applications

**Fig. 4:**
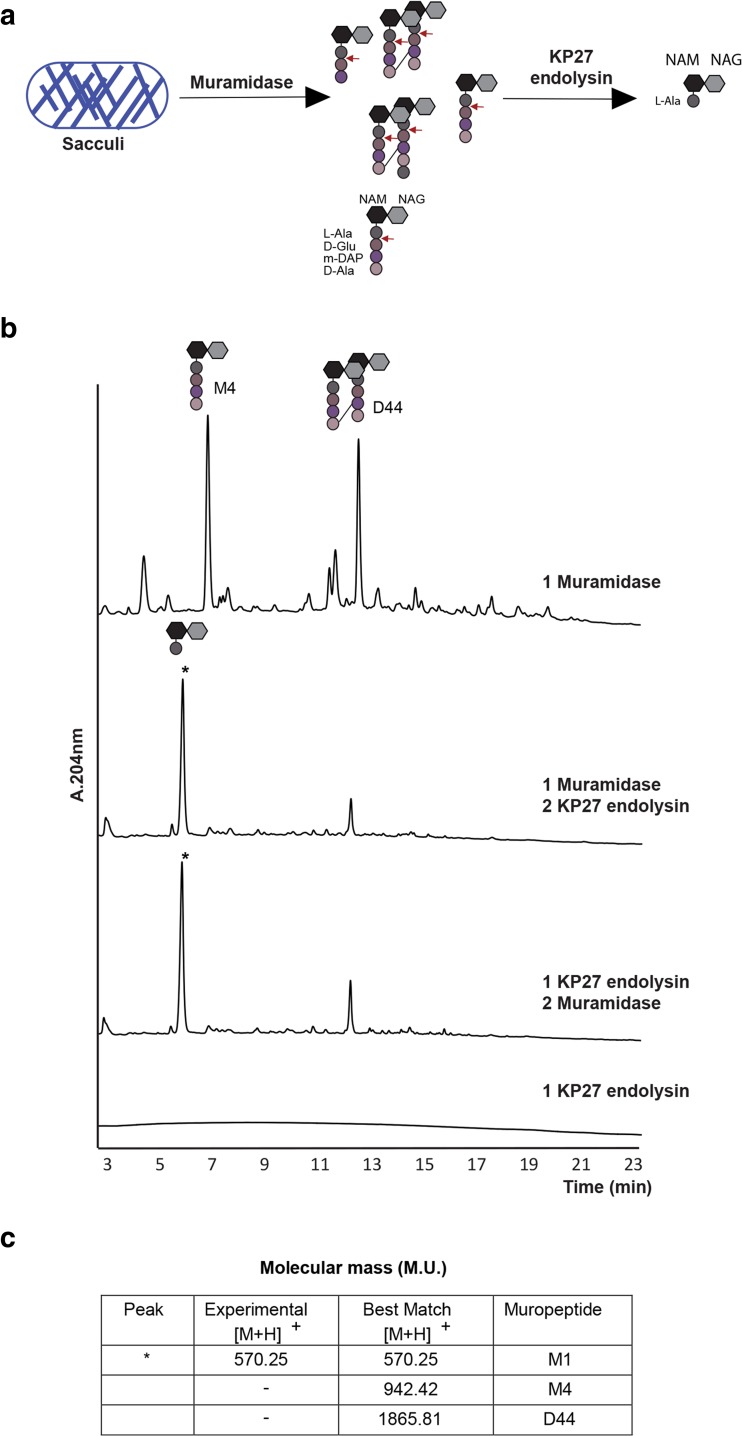
Specific activity of KP27 endolysin. **a** Schematic representation of the solubilized muropeptides after muramidase treatment of *Escherichia coli* murein sacculi. The KP27 endolysin treatment hydrolyzed the peptide bound between l-alanyl-d-glutamate (*red arrows*). *NAM N*-acetyl-muramic acid, *NAG N*-acetyl-glucosamine, *l* *-Ala* l-alanine, *d* *-Glu* d-glutamic acid, *m-DAP* mesodiaminopimelic acid, *d* *-Ala* d-alanine. **b** In vitro endopeptidase assay of KP27 endolysin on *E. coli* sacculi. The *numbers* represent the order in which the reaction was performed. Prior to the reaction with a second enzyme, the individual enzymatic reaction was heat inactivated. *M4* GlcNac-β-(1 → 4)-MurNac-l-Ala-d-Glu-γ-meso-DAP-d-Ala, *D44* cross-linked M4. **c** Mass muropeptide analysis. Experimental MS corresponds to m + z data acquired, while best match corresponds to the theoretical MS value given for each muropeptide. *M1* GlcNac-β-(1 → 4)-MurNac-l-Ala (colour figure online)

To experimentally verify the putative peptidoglycan degrading activity of the enzyme, we produced the recombinant KP27 putative endolysin. The peptidoglycan degrading activity has been confirmed on outer membrane permeabilized bacteria prepared from different Gram-negative species and strains. The enzyme lysed all tested strains, but with different efficiency. For *K. pneumoniae* ATCC 700603 and 486 isolates, a specific activity has been shown of 9580 and 27,360 U/mg respectively, and for *P. aeruginosa* PAO1, *S. enterica* serotype Typhimurium and *E. coli*, a specific activity of 23,700, 17,790 and 17,230 U/mg has been observed respectively. Subsequently, the recombinant enzyme was examined for cleavage specificity by in vitro digestion of purified peptidoglycan. The chromatograms (Fig. 4) show the digestion of *E. coli* peptidoglycan sacculus by the combination of KP27 endolysin and muramidase (Cellosyl, from *Streptomyces coelicolor*, which cleaves on the reducing side of *N*-acetylmuramic acid). The *E. coli* peptidoglycan profile has been establish by Glauner and co-workers (1988) presenting the highest abundance peaks as GlcNac-β-(1 → 4)-MurNac-l-Ala-d-Glu-γ-meso-DAP-d-Ala (M4) and the cross-linked M4 (D44). Addition of KP27 endolysin to muramidase-treated peptidoglycan renders a new peak of 570.25 m.u. This mass is identical to the *N*-acetyl-glucosamine-*N*-acetyl-muramic acid-l-alanine muropeptide (M1) (Fig. 4). Further MS/MS analyses (Fig. S4, Online Resource 1) confirmed the identity of the peak as M1 indicating that KP27 endolysin is a l-alanyl-d-glutamate endopeptidase. The order in which KP27 endolysin and muramidase are added to the peptidoglycan substrate does not affect the UPLC profile, thus indicating that KP27 endolysin can use both soluble muropeptides and undigested peptidoglycan sacculi as substrates. The KP27 endolysins has thus an endopeptidase activity cleaving between l-Ala and d-Glu of the stem peptide and no d-alanyl-d-alanine carboxypeptidase activity as predicted for VanY.

High concentration of the KP27 endopeptidase (5–10 mg/ml) can be stored for 1 month at 4 °C with a loss of maximum 10 %. The endolysin also shows relatively good thermostability, as it does not lose its activity after 1 h incubation at 50, 60 or 70 °C and even after 15 min at 80 °C. An incubation of 60 min at 80 °C results in 10 % loss of its activity, whereas 15 and 60 min incubation at 90 °C causes activity decreases of 35 and 50 % respectively. KP27 endolysin is completely inactivated upon exposure to 110 °C for 30 min. The KP27 endolysin also remains stable for at least 1 h at pH values between 2.6 and 10. Endolysin cytotoxicity evaluation was performed on the epithelial cell line A549 incubated even up to 48 h in the presence of various concentrations of enzyme. No changes in cell viability relative to control cells free of compound were observed, neither in MTT nor in trypan blue exclusion tests determining quantitate cell viability and the CellTox Green Cytotoxicity Assay measuring changes in membrane integrity.

## Discussion

*K. pneumoniae* is dangerous opportunistic pathogen commonly found in hospitals and natural environments. Due to its high virulence and multidrug resistance, *K. pneumoniae* is a common pathogen associated with nosocomial infections that are difficult to combat. The prevalence of those strains among invasive diseases reached up to 60 % according to European Antimicrobial Resistance Surveillance Network reports (http://www.ecdc.europa.eu/en/activities/surveillance/EARS-Net). Nowadays, the interest of the scientific community is more and more focussed on alternative antibacterials such as bacterial predators—bacteriophages and phage-encoded endolysins. There is an increasing number of *Klebsiella* phages propagating on especially extended spectrum beta-lactamase (ESBL) and carbapenemase (KPC) producing isolates (Drulis-Kawa et al. 2011; Kęsik-Szeloch et al. 2013; Mijalis et al. 2015; Provasek et al. 2015; Wangkahad et al. 2015; Jamal et al. 2015), which enables analysis of homology and particular properties of this specific clade of phages. The proposed new Kp15virus genus within the subfamily *Tevenvirinae* groups five phages: *Klebsiella* phages vB_KpnM_KP15 and vB_KpnM_KP27 from the Polish collection; *Klebsiella* phages Matisse and Miro isolated in TX, USA; and *Enterobacter* phage phiEap-3 originating from Beijing, China. All representatives possess high similarity of genome organisation with 95 % identity and high similarity of large subunit terminase proteins. The lysis system composed of holin, antiholin, spanin and endolysin have the same organisation and >99 % protein similarity. The peptidoglycan degrading activity of KP27 endolysin has here been confirmed experimentally. More specifically, we have shown that this endolysin displays l-alanyl-d-glutamate endopeptidase activity, which is in contrast with the in silico predicted d-alanyl-d-alanine carboxypeptidase activity associated with the VanY domain. Similarly, other endolysin encoded by bacteriophage T5, with a predicted d-alanyl-d-alanine carboxypeptidase specificity, has been shown to display l-alanyl-d-glutamate endopeptidase mode of action (Mikoulinskaia et al. 2009). The high stability of the KP27 endolysin tested at different conditions deserves particular attention with regard to its applicability under extreme temperature and pH conditions. Only a few thermostable endolysins have been described so far (Heselpoth et al. 2015; Jin et al. 2013; Lavigne et al. 2004; Matsushita and Yanase 2008; Oliveira et al. 2014; Plotka et al. 2014; Swift et al. 2015). Moreover, KP27 endopeptidase was over three times more potent than HEWL when tested on PAO1 strain (Briers et al. 2007) and no toxicity to human cell line A549 was observed for this enzyme, proving high efficiency and potential safety for application as an antibacterial agent.

The development of molecular biology and novel generation methods of sequencing has opened up new possibilities in preparation of recombinant phage-derived proteins. A special interest is focussed on phage enzymes involved in the first step of viral infection responsible for bacterial envelope degradation, named depolymerases, as well as on proteins encoded by lysis cassette genes such as endolysins (Drulis-Kawa et al. 2015). Although the most studies on endolysin efficacy focus on Gram-positive bacteria, it is already known that these enzymes may be successfully used against Gram-negative representatives, as well (Endersen et al. 2015; Gerstmans et al. 2016). The mixture or fusion with peptides with OM-disrupting properties makes these enzymes permissible though outer membrane barrier reaching the target—the murein. The highly active, thermostable and non-toxic KP27 endopeptidase could find the future application as antimicrobial agents in the fields of medicine, food safety, veterinary, cosmetic and chemical industry, agriculture and biotechnology, against not only *Klebsiella* strains but also other Gram-negative pathogens, as well.

Bacteria can develop phage resistance mechanisms based on several strategies: (i) preventing phage adsorption; (ii) preventing DNA injection by Superinfection Exclusion system (Sie); (iii) degradation of phage DNA by Restriction–Modification (RM) defense system and *Clustered Regularly Interspaced Short Palindromic Repeats* (CRISPR); and (iv) blocking phage replication, transcription, translation or virions assembly by abortive infection system (Abi) (Labrie et al. 2010). Bacterial resistance to phages includes the presence of an active RM system which recognize viral DNA (mostly unmethylated) and can decrease the chance of productive infection but rarely stops infection completely by degradation of viral DNA (Iyer et al. 2013). Most of bacteria and archaea possess restriction–modification systems, in part for defence against DNA bacteriophages, which include two independently active enzymes: a restriction endonuclease (REase) cleaving DNA at a specific sequence target, and a methyltransferase (MTase) modifying the same sequence to protect it from the REase. The activity of these two enzymes must be carefully controlled to ensure protection of the host chromosome. Restriction–modification systems protect the host from foreign invading most often unmodified DNA, such as promiscuous plasmids or infecting bacteriophage. The most common post-replicative base methylations are N6-methyladenine by DNA adenine-N6-methyltransferases (MTases) and 5-methylcytosine modified by cytosine-C5-MTases, which are found in both prokaryotes and eukaryotes, as well in phages as the mechanism of resistance to bacterial RM systems (Malygin et al. 2004; Labrie et al. 2010). Methylation of infectious phage may also confer protection against other host restriction systems or modify the expression of viral genes for expression in other hosts (Smith and Jeddeloh 2005). Concerning potential DNA modification strategies to subvert the host response to viral infection (Labrie et al. 2010), predicted Dcm and Dam methylase encoding genes have been identified in all Kp15virus representatives. However, two unique homing endonucleases (YP_007348875.1 and YP_007348891.1) present in KP27 phage, by splicing event regulate the expression of DNA modification genes located in its close vicinity, resulting broader host range of KP27 phage among *K. pneumoniae* strains in comparison to KP15 phage. It is possible that expression modification of the reductase caused by that regulatory sequence is one of the reasons of KP27 DNA resistance to the restriction digestion. As such, the homing endonucleases of phage KP27 could have an active role which results in the observed difference in DNA susceptibility to restriction enzymes digestion which was previously reported by us (Kęsik-Szeloch et al. 2013). Indeed, KP27 DNA, in contrast to KP15, has been shown to be resistant to numerous type II restriction enzymes including *Eco*RI, *Nsi*I, *Sna*BI or *Eco*RII, even though over 40 recognition sites are present for these particular enzymes. Forthcoming experiments revealing the activity of reductase in possible post-replicative base modification should be carried out. It would be interesting to find out, if the reductase as a recombinant separate enzyme is able to modify DNA molecule, becoming a promising future tool for biotechnology purpose. The interesting aspect is also related to the presence of homing endonucleases in pair with putative DNA-modifying enzyme (reductase), as some kind of controlling element of phage DNA protection strategy. The metagenomics analysis of co-existence of homing endonucleases-reductase within other group of phages could be done to determine the possible presence of novel RM-resistance mechanism developed by bacterial viruses. Our analysis shows that the diversity of *Klebsiella* infecting phages continues to expand. Furthermore, these phages appear suitable (from a molecular microbiology perspective) for possible applications in phage therapy. In addition, these genome analyses serve as a basis for the discovery of relevant proteins and regulatory elements which may find applications for new, enzyme-based antimicrobials and/or as tools for biotechnological applications. Future work may focus on the transcriptional regulation analysis (e.g. using RNAseq) to reveal further fundamental insights into the regulation of the phage infection process, e.g. the elucidation of the role of the homing endonucleases.

## Electronic supplementary material

ESM 1 (PDF 662 kb)
