# Supplementary material for: Klebsiella phages representing a novel clade of viruses with an unknown DNA modification and biotechnologically interesting enzymes
Source: Appl Microbiol Biotechnol. 2016 Oct 21;101(2):673–84. doi: 10.1007/s00253-016-7928-3 (PMC5219037; doi:10.1007/s00253-016-7928-3)
Supplement: Supplementary file 1 — (PDF 662 kb) [file 253_2016_7928_MOESM1_ESM.pdf]

Applied Microbiology and Biotechnology

Klebsiella phages representing a novel clade of viruses with an unknown DNA modification and biotechnologically interesting enzymes

Barbara Maciejewska[1], Bartosz Roszniowski[1], Akbar Espaillat[2], Agata Kęsik-Szeloch[1], Grazyna Majkowska-Skrobek[1], Andrew M. Kropinski[3], Yves Briers[4], Felipe Cava[2], Rob Lavigne[5], and Zuzanna Drulis-Kawa[1*]

[1]Institute of Genetics and Microbiology, University of Wroclaw, Przybyszewskiego 63/77, 51-148 Wroclaw, Poland

[2]Laboratory for Molecular Infection Medicine Sweden. Molecular Biology Department, Umeå University, SE-901 87 Umeå, Sweden

[3]Departments of Food Science, Molecular and Cellular Biology, and Pathobiology, University of Guelph, 50 Stone Road East, Guelph, ON, N1G 2W1, Canada

[4]Department Applied Biosciences, Ghent University, Valentin Vaerwyckweg 1, 9000 Gent, Belgium

[5]Laboratory of Gene Technology, KULeuven, Leuven Kasteelpark Arenberg 21 box 2462, 3001 Leuven, Belgium

*Corresponding author. Mailing address:
Zuzanna Drulis-Kawa
Institute of Genetics and Microbiology, University of Wrocław,
S. Przybyszewskiego 63/77, 51-148 Wrocław, Poland
Tel.: +48 71 375 62 90; Fax: +48 71 325 21 51
E-mail: zuzanna.drulis-kawa@uwr.edu.pl

Table S1a Annotation of KP15 genome

Locus tag	ORF position	Length of product (aa)	Percent identity with homologous proteins from other phages	Predicted molecular function	Characteristic domains
KP-KP15_gp001	1–900	299	100 (*Klebsiella* phage Matisse, *Klebsiella* phage Miro, *Escherichia* phage phT4A)	host membrane ATPase affecting protein	–
KP-KP15_gp002	900–3161	753	100 (*Klebsiella* phage Miro)	RIIA-RIIB membrane-associated protein	pfam13589: HATPase_c_3 (Histidine kinase-, DNA gyrase B-, and HSP90-like ATPase) family
KP-KP15_gp004	4038–5324	428	100 (*Klebsiella* phage Miro)	DNA topoisomerase II medium subunit	pfam00521: DNA_topoisoIV (DNA gyrase/topoisomerase IV, subunit A) family
KP-KP15_gp005	5324–7222	632	100 (*Klebsiella* phage Matisse)	DNA topoisomerase II large subunit	pfam00204: DNA_gyraseB (DNA gyrase B) family
KP-KP15_gp017	11457–12128	223	100 (*Klebsiella* phage KP27, *Klebsiella* phage Matisse, *Enterobacter* phage φEap-3, *Escherichia* phage phT4A)	DexA exonuclease A	PHA02570: dexA (exonuclease)
KP-KP15_gp020	12743–14071	442	99 (*Klebsiella* phage KP27, *Klebsiella* phage Matisse, *Klebsiella* phage Miro, *Enterobacter* phage φEap-3, *Escherichia* phage phT4A)	Dda DNA helicase	pfam13604: AAA_30 (AAA domain) family
KP-KP15_gp022	14540–15076	178	100 (*Klebsiella* phage KP27, *Klebsiella* phage Miro, *Enterobacter* phage φEap-3)	dCTP pyrophosphatase	PHA02602: 56 (dCTP pyrophosphatase)
KP-KP15_gp023	15084–16109	341	99 (*Enterobacter* phage φEap-3)	DNA primase subunit	pfam08275: Toprim_N (DNA primase catalytic core, N-terminal domain) family
KP-KP15_gp026	16644–17132	162	100 (*Klebsiella* phage KP27, *Escherichia* phage phT4A)	gp61.1 hypothetical protein	pfam07068: Gp23 (Major capsid protein Gp23) family

KP-KP15_gp032	19211–20647	478	100 (*Escherichia* phage phT4A)	gp41 replication and recombination DNA helicase	pfam03796: DnaB_C (DnaB-like helicase C terminal domain) family
KP-KP15_gp033	20657–20977	106	100 (*Klebsiella* phage KP27, *Klebsiella* phage Matisse, *Klebsiella* phage Miro, *Enterobacter* phage φEap-3, *Escherichia* phage phT4A)	membrane-associated initiation of head vertex	pfam11113: Phage_head_chap (Head assembly gene product) family
KP-KP15_gp034	21015–22172	385	100 (*Klebsiella* phage Miro, *Escherichia* phage phT4A)	UvsX RecA-like recombination protein	pfam00154: RecA (recA bacterial DNA recombination protein) family
KP-KP15_gp035	22247–24946	899	99 (*Enterobacter* phage φEap-3)	DNA polymerase	pfam03104: DNA_pol_B_exo1 (DNA polymerase family B, exonuclease domain) family; pfam00136: DNA_pol_B (DNA polymerase family B) family
KP-KP15_gp038	25675–26037	120	100 (*Klebsiella* phage Matisse)	RegA translational repressor of early genes	pfam01818: Translat_reg (Bacteriophage translational regulator) family
KP-KP15_gp039	26041–26604	187	99 (*Escherichia* phage phT4A)	gp62 clamp-loader subunit	PHA02593: 62 (clamp loader small subunit)
KP-KP15_gp040	26607–27605	332	100 (*Klebsiella* phage KP27, *Klebsiella* phage Matisse, *Klebsiella* phage Miro, *Enterobacter* phage φEap-3, *Escherichia* phage phT4A)	gp44 clamp-loader subunit	pfam00004: AAA (ATPase family associated with various cellular activities) family
KP-KP15_gp041	27664–28329	221	100 (*Klebsiella* phage KP27, *Klebsiella* phage Matisse, *Klebsiella* phage Miro, *Enterobacter* phage φEap-3, *Escherichia* phage phT4A)	gp45 sliding clamp DNA polymerase	pfam02916: DNA_PPF (DNA polymerase processivity factor) family; pfam09116: gp45-slide_C (gp45 sliding clamp, C terminal) family
KP-KP15_gp042	28356–28625	89	100 (*Klebsiella* phage KP27, *Klebsiella* phage Matisse, *Klebsiella* phage Miro, *Enterobacter* phage φEap-3, *Escherichia* phage phT4A)	RpbA RNA polymerase binding protein	pfam10789: Phage_RpbA (Phage RNA polymerase binding, RpbA) family
KP-KP15_gp043	28681–29652	323	99 (*Escherichia* phage phT4A, *Enterobacter* phage φEap-3)	gp32 ssDNA binding protein	pfam08804: gp32 (gp32 DNA binding protein like) family

KP-KP15_gp044	29668–30246	192	100 (*Klebsiella* phage KP27, *Escherichia* phage phT4A, *Klebsiella* phage Matisse, *Klebsiella* phage Miro, *Enterobacter* phage φEap-3)	gp59 loader of gene 41 DNA helicase	pfam08993: T4_Gp59_N (T4 gene Gp59 loader of gp41 DNA helicase) family; pfam08994: T4_Gp59_C (T4 gene Gp59 loader of gp41 DNA helicase C-term) family
KP-KP15_gp045	30324–30569	81	100 (*Klebsiella* phage KP27, *Klebsiella* phage Matisse, *Klebsiella* phage Miro, *Enterobacter* phage φEap-3, *Escherichia* phage phT4A)	gp33 late promoter transcription	pfam16805: Trans_coact (Phage late-transcription coactivator) family
KP-KP15_gp046	30573–30836	87	100 (*Klebsiella* phage KP27, *Klebsiella* phage Matisse, *Klebsiella* phage Miro, *Escherichia* phage phT4A)	DsbA dsDNA binding	pfam11126: Phage_DsbA (Transcriptional regulator DsbA) family
KP-KP15_gp047	30846–31781	311	100 (*Klebsiella* phage KP27, *Klebsiella* phage Matisse, *Klebsiella* phage Miro, *Enterobacter* phage φEap-3, *Escherichia* phage phT4A)	RnaseH	pfam02739: 5_3_exonuc_N (5'-3' exonuclease, N-terminal resolvase-like domain) family; pfam09293: RNaseH_C (T4 RNase H, C terminal) family
KP-KP15_gp048	32086–32613	175	100 (*Klebsiella* phage KP27, *Klebsiella* phage Matisse, *Klebsiella* phage Miro, *Enterobacter* phage φEap-3)	gp55 RNA polymerase sigma factor	PHA02547: 55 (RNA polymerase sigma factor)
KP-KP15_gp051	33181–34227	348	99 (*Escherichia* phage phT4A)	gp47 recombination endonuclease subunit	PHA02546: 47 (endonuclease subunit)
KP-KP15_gp053	34485–36182	565	99 (*Klebsiella* phage KP27, *Klebsiella* phage Miro)	gp46 recombination endonuclease subunit	pfam13476: AAA_23 (AAA domain) family; pfam13555: AAA_29 (P-loop containing region of AAA domain) family
KP-KP15_gp059	38984–39562	192	99 (*Klebsiella* phage KP27)	Frd dihydrofolate reductase	pfam00186: DHFR_1 (Dihydrofolate reductase) family
KP-KP15_gp060	39564–40439	291	100 (*Klebsiella* phage KP27, *Klebsiella* phage Matisse, *Klebsiella* phage Miro)	Td thymidylate synthetase	pfam00303: Thymidylat_synt (Thymidylate synthase) family
KP-KP15_gp061	40479–42731	750	100 (*Klebsiella* phage Matisse, *Klebsiella* phage Miro, *Enterobacter* phage φEap-3)	NrdA ribonucleotide reductase A subunit	pfam02867: Ribonuc_red_lgC (Ribonucleotide reductase, barrel domain) family

KP-KP15_gp062	42735–43943	402	99 (*Enterobacter* phage φEap-3)	NrdB ribonucleotide reductase B subunit	pfam00268: Ribonuc_red_sm (Ribonucleotide reductase, small chain) family
KP-KP15_gp064	44239–44604	121	100 (*Klebsiella* phage KP27, *Klebsiella* phage Matisse, *Klebsiella* phage Miro, *Enterobacter* phage φEap-3)	DenA endonuclease II	PHA02598: denA (endonuclease II)
KP-KP15_gp065	44585–45742	385	99 (*Klebsiella* phage KP27, *Klebsiella* phage Matisse)	RnlA RNA ligase 1	pfam09511: RNA_lig_T4_1 (RNA ligase) family
KP-KP15_gp066	45739–46062	107	100 (*Klebsiella* phage KP27, *Enterobacter* phage φEap-3)	PseT.3	PRK05846: PRK05846 (NADH:ubiquinone oxidoreductase subunit M)
KP-KP15_gp067	46059–46373	104	100 (*Klebsiella* phage KP27)	PseT.2	–
KP-KP15_gp068	46528–47412	294	99 (*Klebsiella* phage Matisse, *Enterobacter* phage φEap-3)	PseT 3' phosphatase and 5' polynucleotide kinase	pfam13207: AAA_17 (AAA domain) family; pfam13671: AAA_33 (AAA domain) family
KP-KP15_gp072	48216–48773	185	100 (*Escherichia* phage phT4A)	Cd dCMP deaminase	pfam00383: dCMP_cyt_deam_1 (Cytidine and deoxycytidylate deaminase zinc-binding region) family
KP-KP15_gp073	48777–49130	117	100 (*Klebsiella* phage Matisse)	gp31 co-chaperonin for GroEL	pfam00166: Cpn10 (Chaperonin 10 Kd subunit) family
KP-KP15_gp078	51041–51403	120	100 (*Escherichia* phage phT4A)	Vs.6	pfam01228: Gly_radical (Glycine radical) family
KP-KP15_gp079	51403–51672	89	99 (*Klebsiella* phage KP27, *Klebsiella* phage Matisse, *Klebsiella* phage Miro, *Escherichia* phage phT4A)	Vs.4 hypothetical protein	pfam09349: OHCU_decarbox (OHCU decarboxylase) family
KP-KP15_gp088	54307–55836	509	100 (*Klebsiella* phage KP27, *Enterobacter* phage φEap-3, *Escherichia* phage phT4A)	gp30 DNA ligase	PHA00454: PHA00454 (ATP-dependent DNA ligase)
KP-KP15_gp094	57930–59039	369	100 (*Klebsiella* phage Matisse)	gp27 baseplate hub subunit	pfam09097: Phage-tail_1 (Baseplate structural protein, domain 1) family

KP-KP15_gp095	59082–59684	200	100 (*Klebsiella* phage KP27, *Klebsiella* phage Matisse, *Klebsiella* phage Miro, *Escherichia* phage phT4A)	baseplate distal hub subunit	pfam11110: Phage_hub_GP28 (Baseplate hub distal subunit) family
KP-KP15_gp097	61473–62546	357	100 (*Klebsiella* phage KP27)	gp48 baseplate subunit	pfam11091: T4_tail_cap (Tail-tube assembly protein) family
KP-KP15_gp098	62553–63419	288	100 (*Klebsiella* phage Matisse, *Enterobacter* phage φEap-3, *Escherichia* phage phT4A)	gp54 baseplate subunit	PHA02605: 54 (baseplate subunit)
KP-KP15_gp099	63448–64191	247	100 (*Klebsiella* phage KP27, *Klebsiella* phage Matisse, *Klebsiella* phage Miro, *Escherichia* phage phT4A)	gp51 baseplate hub assembly protein	pfam12322: T4_baseplate (T4 bacteriophage base plate protein) family
KP-KP15_gp100	64188–64742	184	100 (*Klebsiella* phage Matisse)	baseplate hub subunit	pfam12322: T4_baseplate (T4 bacteriophage base plate protein) family
KP-KP15_gp101	64743–65135	130	100 (*Klebsiella* phage KP27, *Klebsiella* phage Matisse, *Klebsiella* phage Miro, *Escherichia* phage phT4A)	baseplate wedge subunit	pfam04965: GPW_gp25 (Gene 25-like lysozyme) family
KP-KP15_gp126	78617–78919	100	98 (*Enterobacter* phage φEap-3)	RI membrane protein	PHA02054: PHA02054 (hypothetical protein)
KP-KP15_gp128	79414–80022	202	99 (*Klebsiella* phage KP27)	Tk thymidine kinase	pfam00265: TK (Thymidine kinase) family
KP-KP15_gp130	80495–80968	157	99 (*Klebsiella* phage KP27, *Klebsiella* phage Matisse, *Klebsiella* phage Miro, *Enterobacter* phage φEap-3, *Escherichia* phage phT4A)	Tk.4	pfam01661: Macro (Macro domain) family
KP-KP15_gp136	82957–83598	213	100 (*Klebsiella* phage Matisse, *Klebsiella* phage Miro)	Vs.1	pfam01464: SLT (Transglycosylase SLT domain) family; pfam10715: REGB_T4 (Endoribonuclease RegB T4-bacteriophage encoded) family
KP-KP15_gp140	85820–86344	174	100 (*Klebsiella* phage Matisse)	RB16 HNH(AP2) 1	pfam00847: AP2 (AP2 domain) family

KP-KP15_gp166	97116–97523	135	100 (*Klebsiella* phage KP27, *Klebsiella* phage Matisse, *Klebsiella* phage Miro)	NudE nudix hydrolase	pfam00293: NUDIX (NUDIX domain) family
KP-KP15_gp172	100305–100565	86	100 (*Klebsiella* phage Matisse)	gp57A chaperone for tail fiber formation	–
KP-KP15_gp173	100580–101269	229	99 (*Klebsiella* phage Matisse, *Klebsiella* phage Miro)	deoxynucleoside monophosphate kinase	PHA02575: 1 (deoxynucleoside monophosphate kinase)
KP-KP15_gp174	101269–101799	176	100 (*Klebsiella* phage KP27, *Klebsiella* phage Matisse, *Klebsiella* phage Miro, *Escherichia* phage phT4A)	gp3 tail completion and sheath stabilizer protein	PHA02576: 3 (tail completion and sheath stabilizer protein)
KP-KP15_gp176	102994–103848	284	99 (*Klebsiella* phage KP27, *Enterobacter* phage φEap-3, *Escherichia* phage phT4A)	gp2 DNA end protector protein	PHA02577: 2 (DNA end protector protein)
KP-KP15_gp177	103864–104331	155	100 (*Klebsiella* phage KP27, *Klebsiella* phage Matisse, *Klebsiella* phage Miro, *Enterobacter* phage φEap-3, *Escherichia* phage phT4A)	gp4 head completion protein	PHA02552: 4 (head completion protein)
KP-KP15_gp178	104577–104936	119	99 (*Klebsiella* phage KP27, *Klebsiella* phage Matisse, *Klebsiella* phage Miro, *Escherichia* phage phT4A)	gp53 baseplate wedge subunit	pfam11246: Phage_gp53 (Base plate wedge protein 53) family
KP-KP15_gp179	104933–106702	589	99 (*Klebsiella* phage KP27, *Klebsiella* phage Miro)	gp5 baseplate hub subunit and tail lysozyme	pfam06714: Gp5_OB (Gp5 N-terminal OB domain) family; pfam00959: Phage_lysozyme (Phage lysozyme) family
KP-KP15_gp181	108805–110730	641	99 (*Klebsiella* phage Miro)	gp6 baseplate wedge subunit	PHA02553: 6 (baseplate wedge subunit)
KP-KP15_gp182	110811–113897	1028	99 (*Klebsiella* phage Miro)	gp7 baseplate wedge subunit	PHA02579: 7 (baseplate wedge subunit)
KP-KP15_gp183	113898–114890	330	99 (*Klebsiella* phage Matisse, *Klebsiella* phage Miro, *Enterobacter* phage φEap-3, *Escherichia* phage phT4A)	gp8 baseplate wedge subunit	pfam09215: Phage-Gp8 (Bacteriophage T4, Gp8) family

KP-KP15_gp184	114900–115763	287	100 (*Klebsiella* phage Matisse, *Klebsiella* phage Miro)	gp9 baseplate wedge tail fiber connector	pfam07880: T4_gp9_10 (Bacteriophage T4 gp9/10-like protein) family
KP-KP15_gp185	115760–117577	605	99 (*Klebsiella* phage KP27)	gp10 baseplate wedge subunit and tail pin	pfam07880: T4_gp9_10 (Bacteriophage T4 gp9/10-like protein) family
KP-KP15_gp186	117577–118245	222	100 (*Escherichia* phage phT4A)	gp11 baseplate wedge subunit and tail pin	pfam08677: GP11 (GP11 baseplate wedge protein) family
KP-KP15_gp187	118255–119640	461	100 (*Klebsiella* phage KP27)	gp12 short tail fibers	pfam07484: Collar (Phage Tail Collar Domain) family
KP-KP15_gp188	119654–121405	583	99 (*Klebsiella* phage Miro)	whisker protein	pfam07921: Fibritin_C (Fibritin C-terminal region) family
KP-KP15_gp189	121444–122370	308	100 (*Klebsiella* phage KP27, *Klebsiella* phage Matisse, *Klebsiella* phage Miro)	gp13 neck protein	PHA02554: 13 (neck protein)
KP-KP15_gp190	122380–123126	248	100 (*Klebsiella* phage KP27, *Klebsiella* phage Matisse, *Klebsiella* phage Miro, *Enterobacter* phage φEap-3)	gp14 neck protein	pfam11649: T4_neck-protein (Virus neck protein) family
KP-KP15_gp191	123207–124031	274	100 (*Escherichia* phage phT4A)	gp15 tail sheath stabilizer and completion protein	PHA02556: 15 (tail sheath stabilizer and completion protein)
KP-KP15_gp192	124031–124570	179	100 (*Klebsiella* phage KP27, *Klebsiella* phage Matisse, *Enterobacter* phage φEap-3, *Escherichia* phage phT4A)	gp16 small terminase protein	pfam11053: DNA_Packaging (Terminase DNA packaging enzyme) family
KP-KP15_gp193	124539–126368	609	100 (*Klebsiella* phage KP27)	gp17 large terminase protein	pfam03237: Terminase_6 (Terminase-like) family
KP-KP15_gp194	126388–128379	663	100 (*Klebsiella* phage Matisse)	gp18 tail sheath protein	pfam04984: Phage_sheath_1 (Phage tail sheath protein) family
KP-KP15_gp195	128428–128913	161	100 (*Klebsiella* phage KP27, *Klebsiella* phage Matisse, *Klebsiella* phage Miro,	gp19 tail tube protein	pfam06841: Phage_T4_gp19 (T4-like virus tail tube protein gp19) family

			Enterobacter phage φEap-3, *Escherichia* phage phT4A)		
KP-KP15_gp196	128969–130543	524	100 (*Klebsiella* phage KP27, *Klebsiella* phage Matisse, *Klebsiella* phage Miro, *Enterobacter* phage φEap-3, *Escherichia* phage phT4A)	gp20 portal vertex protein	pfam07230: Phage_T4_Gp20 (Bacteriophage T4-like capsid assembly protein, Gp20) family
KP-KP15_gp197	130543–130785	80	100 (*Klebsiella* phage KP27, *Klebsiella* phage Miro, *Enterobacter* phage φEap-3)	gp67 prohead core protein	PHA02608: 67 (prohead core protein)
KP-KP15_gp198	130794–131198	134	100 (*Klebsiella* phage KP27, *Klebsiella* phage Matisse, *Enterobacter* phage φEap-3, *Escherichia* phage phT4A)	gp68 prohead core protein	PHA02586: 68 (prohead core protein)
KP-KP15_gp199	131201–131848	215	99 (*Klebsiella* phage Matisse, *Enterobacter* phage φEap-3, *Escherichia* phage phT4A)	gp21 prohead core scaffolding protein and protease	pfam03420: Peptidase_S77 (Prohead core protein serine protease) family
KP-KP15_gp200	131881–132669	262	100 (*Klebsiella* phage KP27, *Klebsiella* phage Matisse, *Enterobacter* phage φEap-3, *Escherichia* phage phT4A)	gp22 prohead core protein	PHA02557: 22 (prohead core protein)
KP-KP15_gp201	132689–134257	522	100 (*Klebsiella* phage KP27, *Klebsiella* phage Matisse)	gp23 major capsid protein	pfam07068: Gp23 (Major capsid protein Gp23) family
KP-KP15_gp202	134343–134978	211	99 (*Escherichia* phage phT4A)	SegD homing endonuclease	pfam01541: GIY-YIG (GIY-YIG catalytic domain) family
KP-KP15_gp203	135011–136300	429	99 (*Klebsiella* phage KP27, *Klebsiella* phage Miro, *Escherichia* phage phT4A)	gp24 capsid vertex protein	pfam07068: Gp23 (Major capsid protein Gp23) family
KP-KP15_gp204	136605–137024	139	100 (*Klebsiella* phage KP27, *Klebsiella* phage Matisse, *Klebsiella* phage Miro, *Enterobacter* phage φEap-3, *Escherichia* phage phT4A)	UvsY	pfam11056: UvsY (Recombination, repair and ssDNA binding protein UvsY) family

KP-KP15_gp205	137024–137200	58	100 (*Klebsiella* phage KP27, *Klebsiella* phage Miro, *Enterobacter* phage φEap-3, *Escherichia* phage phT4A)	UvsY.-2	pfam10886: DUF2685 (protein of unknown function DUF2685) family
KP-KP15_gp206	137527–137760	77	100 (*Klebsiella* phage KP27, *Klebsiella* phage Matisse, *Klebsiella* phage Miro, *Enterobacter* phage φEap-3)	UvsW.1 hypothetical protein	pfam11637: UvsW (ATP-dependant DNA helicase UvsW) family
KP-KP15_gp207	137769–139109	446	100 (*Klebsiella* phage KP27, *Klebsiella* phage Matisse, *Klebsiella* phage Miro, *Enterobacter* phage φEap-3, *Escherichia* phage phT4A)	UvsW helicase	pfam04851: ResIII (Type III restriction enzyme, res subunit) family
KP-KP15_gp208	139270–139917	215	100 (*Klebsiella* phage KP27)	Inh minor capsid protein inhibitor of 21 protease	–
KP-KP15_gp211	140959–141492	177	100 (*Klebsiella* phage KP27, *Klebsiella* phage Miro)	Hoc head outer capsid protein	–
KP-KP15_gp212	141503–141778	91	100 (*Klebsiella* phage KP27, *Klebsiella* phage Matisse, *Klebsiella* phage Miro, *Escherichia* phage phT4A)	Hoc large outer capsid protein	–
KP-KP15_gp221	145889–146413	174	99 (*Klebsiella* phage KP27, *Klebsiella* phage Matisse, *Klebsiella* phage Miro, *Enterobacter* phage φEap-3, *Escherichia* phage phT4A)	Srd postulated decoy of host sigma70 or sigmaS	–
KP-KP15_gp222	146417–147436	339	99 (*Klebsiella* phage KP27, *Klebsiella* phage Matisse)	RnlB RNA ligase 2	pfam09414: RNA_ligase (RNA ligase) family
KP-KP15_gp227	149344–150777	477	100 (*Klebsiella* phage KP27, *Klebsiella* phage Matisse, *Enterobacter* phage φEap-3, *Escherichia* phage phT4A)	nicotinamide phosphoribosyl transferase	pfam04095: NAPRTase (Nicotinate phosphoribosyltransferase) family
KP-KP15_gp229	151063–151914	283	99 (*Escherichia* phage phT4A)	Dam DNA adenine methylase	pfam02086: MethyltransfD12 (D12 class N6 adenine-specific DNA methyltransferase) family

KP-KP15_gp230	151923–152198	91	100 (*Klebsiella* phage KP27, *Klebsiella* phage Matisse, *Enterobacter* phage φEap-3, *Escherichia* phage phT4A)	NrdC thioredoxin	pfam00462: Glutaredoxin family
KP-KP15_gp231	152230–152712	160	100 (*Klebsiella* phage KP27, *Enterobacter* phage φEap-3, *Escherichia* phage phT4A)	gp49 recombination endonuclease VII	pfam09124: Endonuc-dimeris (T4 recombination endonuclease VII, dimerisation) family
KP-KP15_gp232	152709–154832	707	99 (*Enterobacter* phage φEap-3)	NrdD anaerobic ribonucleotide reductase subunit	pfam03477: ATP-cone (ATP cone domain) family; pfam01228: Gly_radical (Glycine radical) family
KP-KP15_gp240	157386–157877	163	99 (*Enterobacter* phage φEap-3)	NrdG anaerobic nucleotide reductase subunit	pfam13353: Fer4_12 (4Fe-4S single cluster domain) family; pfam13394: Fer4_14 (4Fe-4S single cluster domain) family
KP-KP15_gp243	159577–159858	93	99 (*Klebsiella* phage KP27, *Klebsiella* phage Matisse, *Klebsiella* phage Miro, *Enterobacter* phage φEap-3, *Escherichia* phage phT4A)	NrdH	pfam00462: Glutaredoxin family
KP-KP15_gp245	160234–164007	1257	99 (*Klebsiella* phage Miro)	gp34 long tail fiber proximal subunit	PHA02584: 34 (long tail fiber, proximal subunit)
KP-KP15_gp246	164007–165131	374	99 (*Klebsiella* phage KP27)	gp35 hinge connector of long tail fiber proximal connector	–
KP-KP15_gp247	165181–165849	222	95 (*Klebsiella* phage Matisse)	hinge connector of long tail fiber distal connector	pfam03903: Phage_T4_gp36 (Phage T4 tail fibre) family
KP-KP15_gp248	165858–169844	1328	80 (*Escherichia* phage phT4A)	L-shaped tail fiber protein	pfam13884: Peptidase_S74 (Chaperone of endosialidase) family
KP-KP15_gp249	169881–170408	175	99 (*Klebsiella* phage Miro)	gp38 distal long tail fiber assembly catalyst	pfam05268: GP38 (Phage tail fibre adhesin Gp38) family
KP-KP15_gp250	170525–171172	215	100 (*Klebsiella* phage Matisse, *Klebsiella* phage Miro)	T holin	pfam11031: Phage_holin_T (Bacteriophage T holin) family

KP-KP15_gp256	173605–174042	145	100 (*Klebsiella* phage Miro, *Enterobacter* phage φEap-3)	Ndd	–

Table S1b Annotation of KP27 genome

Locus tag	ORF position	Length of product (aa)	Percent identity with homologous proteins from other phages	Predicted molecular function	Characteristic domains
G376_gp276	1–2262	753	99 (*Klebsiella* phage KP15, *Klebsiella* phage Miro)	rIIA protector from prophage-induced early lysis	pfam13589: HATPase_c_3 (Histidine kinase-, DNA gyrase B-, and HSP90-like ATPase) family
G376_gp273	3139–4425	428	100 (*Klebsiella* phage Matisse)	DNA topoisomerase medium subunit	pfam00521: DNA_topoisoIV (DNA gyrase/topoisomerase IV, subunit A) family
G376_gp272	4425–6323	632	100 (*Klebsiella* phage Miro)	DNA topoisomerase large subunit	pfam00204: DNA_gyraseB (DNA gyrase B) family
G376_gp257	10819–11490	223	100 (*Klebsiella* phage KP15, *Klebsiella* phage Matisse, *Enterobacter* phage φEap-3, *Escherichia* phage phT4A)	exonuclease A	PHA02570: dexA (exonuclease)
G376_gp254	12105–13433	442	100 (*Klebsiella* phage Matisse, *Klebsiella* phage Miro, *Enterobacter* phage φEap-3, *Escherichia* phage phT4A)	DNA helicase	pfam13604: AAA_30 (AAA domain) family
G376_gp251	13902–14438	178	100 (*Klebsiella* phage KP15, *Klebsiella* phage Miro, *Enterobacter* phage φEap-3)	dCTPase	PHA02602: 56 (dCTP pyrophosphatase)
G376_gp250	14446–15471	341	100 (*Klebsiella* phage Matisse, *Klebsiella* phage Miro, *Escherichia* phage phT4A)	DNA primase subunit	pfam08275: Toprim_N (DNA primase catalytic core, N-terminal domain) family

G376_gp241	18576–20012	478	100 (*Klebsiella* phage Matisse, *Klebsiella* phage Miro)	replication and recombination DNA helicase	pfam03796: DnaB_C (DnaB-like helicase C terminal domain) family
G376_gp240	20022–20342	106	100 (*Klebsiella* phage KP15, *Klebsiella* phage Matisse, *Klebsiella* phage Miro, *Enterobacter* phage φEap-3, *Escherichia* phage phT4A)	head vertex assembly chaperone	pfam11113: Phage_head_chap (Head assembly gene product) family
G376_gp239	20378–21535	385	99 (*Klebsiella* phage KP15, *Klebsiella* phage Miro, *Escherichia* phage phT4A)	RecA-like recombination protein	pfam00154: RecA (recA bacterial DNA recombination protein) family
G376_gp238	21610–24309	899	99 (*Klebsiella* phage Matisse)	DNA polymerase	pfam03104: DNA_pol_B_exo1 (DNA polymerase family B, exonuclease domain) family; pfam00136: DNA_pol_B (DNA polymerase family B) family
G376_gp235	25038–25400	120	100 (*Klebsiella* phage Miro, *Enterobacter* phage φEap-3, *Escherichia* phage phT4A)	RegA	pfam01818: Translat_reg (Bacteriophage translational regulator) family
G376_gp234	25404–25967	187	99 (*Klebsiella* phage KP15)	clamp loader small subunit	PHA02593: 62 (clamp loader small subunit)
G376_gp233	25970–26968	332	100 (*Klebsiella* phage KP15, *Klebsiella* phage Matisse, *Klebsiella* phage Miro, *Enterobacter* phage φEap-3, *Escherichia* phage phT4A)	clamp-loader subunit	pfam00004: AAA (ATPase family associated with various cellular activities) family
G376_gp232	27027–27692	221	100 (*Klebsiella* phage KP15, *Klebsiella* phage Matisse, *Klebsiella* phage Miro, *Enterobacter* phage φEap-3, *Escherichia* phage phT4A)	sliding clamp protein	pfam02916: DNA_PPF (DNA polymerase processivity factor) family; pfam09116: gp45-slide_C (gp45 sliding clamp, C terminal) family
G376_gp231	27719–27988	89	100 (*Klebsiella* phage KP15, *Klebsiella* phage Matisse, *Klebsiella* phage Miro, *Enterobacter* phage φEap-3, *Escherichia* phage phT4A)	RNA polymerase binding protein	pfam10789: Phage_RpbA (Phage RNA polymerase binding, RpbA) family
G376_gp230	28044–29015	323	100 (*Klebsiella* phage Matisse)	ssDNA binding protein	pfam08804: gp32 (gp32 DNA binding protein like) family

G376_gp229	29031–29690	219	99 (*Klebsiella* phage Matisse, *Klebsiella* phage Miro, *Enterobacter* phage φEap-3)	loader of DNA helicase	pfam08993: T4_Gp59_N (T4 gene Gp59 loader of gp41 DNA helicase) family; pfam08994: T4_Gp59_C (T4 gene Gp59 loader of gp41 DNA helicase C-term) family
G376_gp228	29687–29932	81	100 (*Klebsiella* phage KP15, *Klebsiella* phage Matisse, *Klebsiella* phage Miro, *Enterobacter* phage φEap-3, *Escherichia* phage phT4A)	late promoter transcription accessory protein	pfam16805: Trans_coact (Phage late-transcription coactivator) family
G376_gp227	29936–30199	87	100 (*Klebsiella* phage KP15, *Klebsiella* phage Matisse, *Klebsiella* phage Miro, *Escherichia* phage phT4A)	dsDNA binding protein late transcription	pfam11126: Phage_DsbA (Transcriptional regulator DsbA) family
G376_gp226	30209–31144	311	100 (*Klebsiella* phage KP15, *Klebsiella* phage Matisse, *Klebsiella* phage Miro, *Enterobacter* phage φEap-3, *Escherichia* phage phT4A)	RNaseH ribonuclease	pfam02739: 5_3_exonuc_N (5'-3' exonuclease, N-terminal resolvase-like domain) family; pfam09293: RNaseH_C (T4 RNase H, C terminal) family
G376_gp225	31449–31976	175	100 (*Klebsiella* phage KP15, *Klebsiella* phage Matisse, *Klebsiella* phage Miro, *Enterobacter* phage φEap-3)	sigma factor for late transcription	PHA02547: 55 (RNA polymerase sigma factor)
G376_gp222	32559–33590	343	99 (*Escherichia* phage phT4A)	recombination endonuclease subunit	PHA02546: 47 (endonuclease subunit)
G376_gp220	33848–35545	565	100 (*Klebsiella* phage Miro)	recombination endonuclease subunit	pfam13476: AAA_23 (AAA domain) family; pfam13555: AAA_29 (P-loop containing region of AAA domain) family
G376_gp214	38347–38925	192	99 (*Klebsiella* phage KP15)	dihydrofolate reductase	pfam00186: DHFR_1 (Dihydrofolate reductase) family
G376_gp213	38927–39802	291	100 (*Klebsiella* phage KP15, *Klebsiella* phage Matisse, *Klebsiella* phage Miro)	thymidylate synthetase	pfam00303: Thymidylat_synt (Thymidylate synthase) family
G376_gp212	39842–42094	750	99 (*Klebsiella* phage KP15, *Klebsiella* phage Matisse, *Klebsiella* phage Miro, *Enterobacter* phage φEap-3)	aerobic ribonucleotide reductase large subunit	pfam02867: Ribonuc_red_lgC (Ribonucleotide reductase, barrel domain) family
G376_gp211	42131–43306	391	100 (*Klebsiella* phage Matisse, *Klebsiella* phage Miro)	aerobic ribonucleotide reductase B subunit	pfam00268: Ribonuc_red_sm (Ribonucleotide reductase, small chain) family

G376_gp209	43873–44238	121	100 (*Klebsiella* phage KP15, *Klebsiella* phage Matisse, *Klebsiella* phage Miro, *Enterobacter* phage φEap-3)	endonuclease II	PHA02598: denA (endonuclease II)
G376_gp208	44219–45376	385	100 (*Klebsiella* phage Matisse)	RNA ligase 1 and tail fiber attachment catalyst	pfam09511: RNA_lig_T4_1 (RNA ligase) family
G376_gp204	46162–47046	294	99 (*Klebsiella* phage Matisse, *Enterobacter* phage φEap-3)	3' phosphatase, 5' polynucleotide kinase	pfam13207: AAA_17 (AAA domain) family; pfam13671: AAA_33 (AAA domain) family
G376_gp200	47901–48407	168	100 (*Enterobacter* phage φEap-3)	dCMP deaminase	pfam00383: dCMP_cyt_deam_1 (Cytidine and deoxycytidylate deaminase zinc-binding region) family
G376_gp199	48423–48764	113	100 (*Enterobacter* phage φEap-3, *Escherichia* phage phT4A)	co-chaperonin for GroEL	pfam00166: Cpn10 (Chaperonin 10 Kd subunit) family
G376_gp183	53940–55469	509	100 (*Klebsiella* phage KP15, *Enterobacter* phage φEap-3, *Escherichia* phage phT4A)	gp30 DNA ligase	PHA00454: PHA00454 (ATP-dependent DNA ligase)
G376_gp177	56924–58033	369	100 (*Escherichia* phage phT4A)	baseplate hub subunit	pfam09097: Phage-tail_1 (Baseplate structural protein, domain 1) family
G376_gp176	58103–58678	191	100 (*Klebsiella* phage KP15, *Klebsiella* phage Matisse, *Klebsiella* phage Miro, *Escherichia* phage phT4A)	baseplate distal hub subunit	pfam11110: Phage_hub_GP28 (Baseplate hub distal subunit) family
G376_gp175	58681–60456	591	100 (*Klebsiella* phage Matisse)	putative base plate hub	–
G376_gp174	60467–61540	357	100 (*Klebsiella* phage KP15)	baseplate tail tube cap	pfam11091: T4_tail_cap (Tail-tube assembly protein) family
G376_gp173	61550–62413	287	100 (*Klebsiella* phage Miro)	baseplate-tail tube initiator	PHA02605: 54 (baseplate subunit)

G376_gp172	62442–63185	247	100 (*Klebsiella* phage KP15, *Klebsiella* phage Matisse, *Klebsiella* phage Miro, *Escherichia phage* phT4A)	baseplate hub assembly catalyst	pfam12322: T4_baseplate (T4 bacteriophage base plate protein) family
G376_gp171	63182–63736	184	100 (*Klebsiella* phage Miro, *Enterobacter* phage φEap-3, *Escherichia* phage phT4A)	baseplate hub subunit	pfam12322: T4_baseplate (T4 bacteriophage base plate protein) family
G376_gp170	63737–64129	130	100 (*Klebsiella* phage KP15, *Klebsiella* phage Matisse, *Klebsiella* phage Miro, *Escherichia* phage phT4A)	baseplate wedge subunit	pfam04965: GPW_gp25 (Gene 25-like lysozyme) family
G376_gp154	71150–72061	303	99 (*Klebsiella* phage Matisse)	cytosine-specific methyltransferase	pfam00145: DNA_methylase (C-5 cytosine-specific DNA methylase) family
G376_gp142	78026–78307	93	99 (*Enterobacter* phage φEap-3)	rI lysis inhibition regulator	PHA02054: PHA02054 (hypothetical protein)
G376_gp140	78823–79431	202	99 (*Klebsiella* phage Matisse)	thymidine kinase	pfam00265: TK (Thymidine kinase) family
G376_gp128	83534–84883	449	99 (*Enterobacter* phage φEap-3)	putative PhoH family protein	pfam02562: PhoH (PhoH-like protein) family
G376_gp127	84892–85416	174	57 (*Enterobacteria* phage RB16)	putative homing endonuclease	pfam00847: AP2 (AP2 domain) family
G376_gp099	95748–96155	135	100 (*Klebsiella* phage KP15, *Klebsiella* phage Matisse, *Klebsiella* phage Miro)	nudix hydrolase	pfam00293: NUDIX (NUDIX domain) family
G376_gp090	98936–99196	86	100 (*Klebsiella* phage Miro)	chaperone for tail fiber formation	–
G376_gp089	99211–99906	231	99 (*Klebsiella* phage Matisse, *Klebsiella* phage Miro)	deoxynucleoside monophosphate kinase	PHA02575: 1 (deoxynucleoside monophosphate kinase)

G376_gp088	99906–100436	176	100 (*Klebsiella* phage KP15, *Klebsiella* phage Matisse, *Klebsiella* phage Miro, *Escherichia* phage phT4A)	head-proximal tip of tail tube	PHA02576: 3 (tail completion and sheath stabilizer protein)
G376_gp086	101631–102485	284	100 (*Enterobacter* phage φEap-3, *Escherichia* phage phT4A)	DNA end protector protein	PHA02577: 2 (DNA end protector protein)
G376_gp085	102501–102968	155	100 (*Klebsiella* phage KP15, *Klebsiella* phage Matisse, *Klebsiella* phage Miro, *Enterobacter* phage φEap-3, *Escherichia* phage phT4A)	head completion protein	PHA02552: 4 (head completion protein)
G376_gp084	103019–103573	184	100 (*Klebsiella* phage Matisse, *Klebsiella* phage Miro, *Escherichia* phage phT4A)	baseplate wedge subunit	pfam11246: Phage_gp53 (Base plate wedge protein 53) family
G376_gp083	103570–105339	589	100 (*Klebsiella* phage Miro)	baseplate hub subunit and tail lysozyme	pfam06714: Gp5_OB (Gp5 N-terminal OB domain) family; pfam00959: Phage_lysozyme (Phage lysozyme) family
G376_gp081	107442–109367	641	99 (*Enterobacter* phage φEap-3)	baseplate wedge subunit	PHA02553: 6 (baseplate wedge subunit)
G376_gp080	109448–112534	1028	99 (*Klebsiella* phage Matisse)	baseplate wedge subunit	PHA02579: 7 (baseplate wedge subunit)
G376_gp079	112535–113527	330	99 (*Klebsiella* phage Matisse, *Klebsiella* phage Miro, *Enterobacter* phage φEap-3, *Escherichia* phage phT4A)	baseplate wedge subunit	pfam09215: Phage-Gp8 (Bacteriophage T4, Gp8) family
G376_gp078	113537–114400	287	100 (*Enterobacter* phage φEap-3)	baseplate wedge tail fiber connector	pfam07880: T4_gp9_10 (Bacteriophage T4 gp9/10-like protein) family
G376_gp077	114397–116214	605	99 (*Escherichia* phage phT4A)	baseplate wedge subunit and tail pin	pfam07880: T4_gp9_10 (Bacteriophage T4 gp9/10-like protein) family
G376_gp076	116214–116882	222	99 (*Klebsiella* phage KP15, *Escherichia* phage phT4A)	baseplate wedge subunit tail pin	pfam08677: GP11 (GP11 baseplate wedge protein) family

G376_gp075	116892–118277	461	100 (*Klebsiella* phage KP15)	short tail fibers	pfam07484: Collar (Phage Tail Collar Domain) family
G376_gp074	118288–120039	583	99 (*Enterobacter* phage φEap-3)	fibritin neck whiskers	pfam07921: Fibritin_C (Fibritin C-terminal region) family
G376_gp073	120078–121004	308	100 (*Klebsiella* phage KP15, *Klebsiella* phage Matisse, *Klebsiella* phage Miro)	head completion	PHA02554: 13 (neck protein)
G376_gp072	121014–121760	248	100 (*Klebsiella* phage KP15, *Klebsiella* phage Matisse, *Klebsiella* phage Miro, *Enterobacter* phage φEap-3)	head completion	pfam11649: T4_neck-protein (Virus neck protein) family
G376_gp071	121840–122664	274	99 (*Klebsiella* phage Matisse)	tail sheath stabilizer and completion protein	PHA02556: 15 (tail sheath stabilizer and completion protein)
G376_gp070	122664–123203	179	100 (*Klebsiella* phage KP15, *Klebsiella* phage Matisse, *Enterobacter* phage φEap-3, *Escherichia* phage phT4A)	small terminase protein	pfam11053: DNA_Packaging (Terminase DNA packaging enzyme) family
G376_gp069	123172–125001	609	100 (*Klebsiella* phage KP15)	terminase subunit nuclease and ATPase	pfam03237: Terminase_6 (Terminase-like) family
G376_gp068	125021–127012	663	99 (*Klebsiella* phage KP15, *Klebsiella* phage Matisse)	tail sheath protein	pfam04984: Phage_sheath_1 (Phage tail sheath protein) family
G376_gp067	127061–127546	161	100 (*Klebsiella* phage KP15, *Klebsiella* phage Matisse, *Klebsiella* phage Miro, *Enterobacter* phage φEap-3, *Escherichia* phage phT4A)	tail tube monomer	pfam06841: Phage_T4_gp19 (T4-like virus tail tube protein gp19) family
G376_gp066	127602–129176	524	100 (*Klebsiella* phage KP15, *Klebsiella* phage Matisse, *Klebsiella* phage Miro, *Enterobacter* phage φEap-3, *Escherichia* phage phT4A)	portal vertex of the head	pfam07230: Phage_T4_Gp20 (Bacteriophage T4-like capsid assembly protein, Gp20) family
G376_gp065	129176–129418	80	100 (*Klebsiella* phage KP15, *Klebsiella* phage Miro, *Enterobacter* phage φEap-3)	prohead core protein	PHA02608: 67 (prohead core protein)

G376_gp064	129427–129831	134	100 (*Klebsiella* phage KP15, *Klebsiella* phage Matisse, *Enterobacter* phage φEap-3, *Escherichia* phage phT4A)	prohead core protein	PHA02586: 68 (prohead core protein)
G376_gp063	129834–130481	215	99 (*Klebsiella* phage Matisse, *Enterobacter* phage φEap-3, *Escherichia* phage phT4A)	prohead core and protease	pfam03420: Peptidase_S77 (Prohead core protein serine protease) family
G376_gp062	130514–131302	262	100 (*Klebsiella* phage KP15, *Klebsiella* phage Matisse, *Enterobacter* phage φEap-3, *Escherichia* phage phT4A)	prohead core protein	PHA02557: 22 (prohead core protein)
G376_gp061	131322–132890	522	100 (*Klebsiella* phage KP15, *Klebsiella* phage Matisse)	major capsid protein	pfam07068: Gp23 (Major capsid protein Gp23) family
G376_gp060	132982–133620	212	99 (*Klebsiella* phage Matisse)	homing endonuclease	pfam01541: GIY-YIG (GIY-YIG catalytic domain) family
G376_gp059	133653–134942	429	100 (*Klebsiella* phage Miro, *Escherichia* phage phT4A)	capsid vertex protein	pfam07068: Gp23 (Major capsid protein Gp23) family
G376_gp058	135256–135675	139	100 (*Klebsiella* phage KP15, *Klebsiella* phage Matisse, *Klebsiella* phage Miro, *Enterobacter* phage φEap-3, *Escherichia* phage phT4A)	recombination, repair and ssDNA binding protein	pfam11056: UvsY (Recombination, repair and ssDNA binding protein UvsY) family
G376_gp055	136368–137867	499	100 (*Klebsiella* phage Matisse, *Klebsiella* phage Miro, *Enterobacter* phage φEap-3, *Escherichia* phage phT4A)	helicase	pfam04851: ResIII (Type III restriction enzyme, res subunit) family
G376_gp054	137938–138516	192	100 (*Klebsiella* phage KP15)	minor capsid protein inhibitor of protease	–
G376_gp051	139558–140091	177	100 (*Klebsiella* phage KP15, *Klebsiella* phage Miro)	Hoc head outer capsid protein	–
G376_gp050	140102–140377	91	100 (*Klebsiella* phage KP15, *Klebsiella* phage Matisse, *Klebsiella* phage Miro, *Escherichia* phage phT4A)	Hoc large outer capsid protein	–

G376_gp041	144488–145012	174	100 (*Klebsiella* phage Matisse, *Klebsiella* phage Miro, *Enterobacter* phage φEap-3, *Escherichia* phage phT4A)	postulated decoy of host sigma70 or sigmaS	–
G376_gp040	145016–146035	339	100 (*Klebsiella* phage Matisse)	RNA ligase 2	pfam09414: RNA_ligase (RNA ligase) family
G376_gp039	146039–146557	172	54 (*Enterobacteria* phage RB16)	putative homing endonuclease	pfam13392: HNH_3 (HNH endonuclease) family
G376_gp034	148471–149904	477	100 (*Klebsiella* phage KP15, *Klebsiella* phage Matisse, *Enterobacter* phage φEap-3, *Escherichia* phage phT4A)	putative nicotinate phosphoribosyltransferase	pfam04095: NAPRTase (Nicotinate phosphoribosyltransferase) family
G376_gp032	150190–151041	283	99 (*Escherichia* phage phT4A)	DNA adenine methylase	pfam02086: MethyltransfD12 (D12 class N6 adenine-specific DNA methyltransferase) family
G376_gp031	151050–151325	91	100 (*Klebsiella* phage KP15, *Klebsiella* phage Matisse, *Enterobacter* phage φEap-3, *Escherichia* phage phT4A)	thioredoxin	pfam00462: Glutaredoxin family
G376_gp030	151357–151839	160	100 (*Klebsiella* phage KP15, *Enterobacter* phage φEap-3, *Escherichia* phage phT4A)	recombination endonuclease VII	pfam09124: Endonuc-dimeris (T4 recombination endonuclease VII, dimerisation) family
G376_gp029	151836–153959	707	99 (*Enterobacter* phage φEap-3)	anaerobic ribonucleotide reductase subunit	pfam03477: ATP-cone (ATP cone domain) family; pfam01228: Gly_radical (Glycine radical) family
G376_gp023	155517–156158	213	99 (*Klebsiella* phage Matisse, *Klebsiella* phage Miro)	putative homing endonuclease	pfam01844: HNH (HNH endonuclease) family
G376_gp022	156155–156646	163	100 (*Klebsiella* phage Miro)	anaerobic nucleotide reductase subunit	pfam13353: Fer4_12 (4Fe-4S single cluster domain) family; pfam13394: Fer4_14 (4Fe-4S single cluster domain) family
G376_gp019	158346–158627	93	100 (*Klebsiella* phage Matisse, *Klebsiella* phage Miro, *Enterobacter* phage φEap-3, *Escherichia* phage phT4A)	glutaredoxin	pfam00462: Glutaredoxin family

G376_gp017	159003–162776	1257	99 (*Klebsiella* phage Matisse)	proximal tail fiber subunit	PHA02584: 34 (long tail fiber, proximal subunit)
G376_gp016	162776–163900	374	99 (*Klebsiella* phage Matisse)	hinge connector of long tail fiber proximal connector	–
G376_gp015	163950–164618	222	99 (*Klebsiella* phage Miro, *Enterobacter* phage φEap-3)	hinge connector of long tail fiber distal connector	pfam03903: Phage_T4_gp36 (Phage T4 tail fibre) family
G376_gp014	164627–168700	1357	86 (*Klebsiella* phage Matisse)	L-shaped tail fiber protein	pfam13884: Peptidase_S74 (Chaperone of endosialidase) family
G376_gp013	168740–169267	175	98 (*Klebsiella* phage KP15)	distal long tail fiber assembly catalyst	pfam05268: GP38 (Phage tail fibre adhesin Gp38) family
G376_gp012	169379–170026	215	99 (*Klebsiella* phage KP15, *Klebsiella* phage Matisse, *Klebsiella* phage Miro)	T holin lysis mediator	pfam11031: Phage_holin_T (Bacteriophage T holin) family
G376_gp003	172683–173063	126	100 (*Escherichia* phage phT4A)	nucleoid disruption protein	–
G376_gp001	173515–174405	296	99 (*Klebsiella* phage KP15, *Klebsiella* phage Matisse, *Klebsiella* phage Miro, *Escherichia* phage phT4A)	rIIB protein	–

Table S2 KP15 phage predicted promoters, terminators and tRNA requences

**Putative host promoters of KP15**		
Location		Sequence
Start	End	
10075	10042	**TTGTTA**AAACATTCTGGGGTAGCTTTGG**TATAGT**
10425	10386	**AAAGAA**GCCTCTTGCATTGCGGGAGGCTTTTTG**GTATCT**
11177	11136	**AAAACA**AAGCCCTTGACTTCGGTTGAGGGCTTTTGT**TATATT**
18890	18863	**TTGACC**TGAATCTCCATTGTGG**TAATAT**
25663	25635	**TTGCAT**TCGTGCAGGGGCTTTGT**TATATT**
55838	55875	**AAAAA**GCCCTTGACTCCGGTCGGGGGCTTTGT**TATATT**
56495	56543	**AAAGA**AGCCTCTTGCAATGCAGGGGGCTTTTTTG**TATATT**
56848	56876	**TTGTGT**TCTGGGTAATGTAATGG**TACATT**
75902	75862	**AAAAA**GCCCTTGCGTTCTCGTGAGGGCTTTTG**TATTAT**
82955	82918	**AAAAA**GCCCTTGACTTCGGTCGGGGGCTTTTT**TATTAT**
88440	88413	**TTGACA**CTAATCCCCGTTGTGA**TAATAT**
91788	91760	**TTGTGT**TTGGTTCTTTATAGGGT**TATATT**
92611	92581	**TTGCTA**AAACTCGATAACCCTGATC**TGTTAT**
92975	92938	**TTGCCT**ATGGTGAGGGGTTTTGT**TATATT**
98843	98814	**TGTTGA**CTTTGGTTTATATTATGG**TAAGAT**
148527	148555	**TTGACG**GGGGTTGGTTATAGTAG**TATTCT**
54835	154873	**TTGACT**TCGGTTGAGGGCTTTTGT**TATATT**
**Rho-independent terminators of KP15**		
Location		Sequence
Start	End	
7288	7277	CCCCGAAAGGGG
9393	9382	GGGCTTCGGCCC
10419	10399	GCCTCTTGCATTGCGGGAGGC

11168	11149	CCCTTGACTTCGGTTGAGGG
18556	18543	GCCCCGAAAGGGGC
18543	18556	GCCCCTTTCGGGGC
20991	21010	TATGGGGCCGAAGCCCCATA
22240	22227	GGGGCTTCGGCCCC
25667	25646	GCCCTTGCATTCGTGCAGGGGC
27652	27639	GGGGCTACGGCCCC
28656	28675	GTGGGGGCCGAAGCCCCCGC
36400	36421	GCCCATGTAACGAAACGTGGGC
40445	40458	GGGGCTTCGGCCCC
50429	50446	GGCTATCTTCGGATAGCC
51680	51701	GCCGTTGCCGAAAGGTAGCGGC
55843	55864	GCCCTTGACTCCGGTCGGGGGC
63421	63434	GGGGCTACGGCCCC
65142	65163	GCCCCTTCCTTTCGGTTGGGGC
65163	65142	GCCCCAACCGAAAGGAAGGGGC
71395	71382	GCCCCGAAAGGGGC
75894	75873	GCCCTTGCGTTCTCGTGAGGGC
75933	75962	CCGCACATAAAAGATCATCTATATGTGCGG
82949	82930	CCCTTGACTTCGGTCGGGGG
84459	84444	GGGGGCGAAAGCCCCC
88102	88089	GCCCCGAAAGGGGC
92970	92950	CCCCTTGCCTATGGTGAGGGG
94665	94644	GCCCTTGCATTAGTGCAGGGGC
99077	99056	GCCCTTGCATTCGTGCAGGGGC
100286	100273	GGGGCTACGGCCCC
102986	102965	GCCCCAACCGAAAGGAAGGGGC
102965	102986	GCCCCTTCCTTTCGGTTGGGGC
110738	110757	CCCCTGTCTTCGGATAGGGG

121416	121429	GGGGCTTCGGCCCC
128928	128943	GGGGTAGCAATACCCC
134283	134300	GGGAACCTTCGGGTTCCC
134300	134283	GGGAACCCGAAGGTTCCC
136335	136360	GGTGGAGTAGGGAAACTTACTCCATC
137521	137500	GCCCCAACCGAAAGGAAGGGGC
137500	137521	GCCCCTTCCTTTCGGTTGGGGC
140329	140342	GGGGCTACGGCCCC
142335	142317	GGGAACCATTGCGGTTCCC
142317	142335	GGGAACCGCAATGGTTCCC
147481	147460	CGGCTACCGATCAGGGTAGCCG
154840	154861	GCCCTTGACTTCGGTTGAGGGC
158915	158930	GGGTGCTCCGGCACCC
160171	160184	TGGCCTTCGGGCCA
165137	165154	GGCCCGGAACATCGGGCC
165154	165137	GGCCCGATGTTCCGGGCC
169850	169863	GGGGCTTCGGCCCC
170454	170472	GGCTACCATATAGGTAGCC
170472	170454	GGCTACCTATATGGTAGCC

tRNA of KP15

Location		Sequence
Start	End	
49401	49477	GGCCCCTTAGCTCAATAGGTAGAGCTAATCACTCATAATGGTTAGGTTCCCGGTTCAAGTCACGGGAGGGGCCACCA
49483	49559	GGCAAGGCACTTAGAGAAGCGGGAGGAACAGAATCTGATTCTGTTGGTTGTAGGTGCAAATCCTACCCTTGCCACCA

Table S3 KP27 phage predicted promoters, terminators and tRNA requences

**Putative host promoters of KP27**		
Location		Sequence
Start	End	
3126	3098	**TTGCAA**TGCAGGGGGCTTTTTGC**TATATT**
8896	8868	**TTGCAT**AGAATAACCATCTTTGA**TATAGT**
9178	9144	**TTGCTA**AAGACTTCACTGGGTAGCTTTGG**TATAGT**
9778	9750	**TTGCAT**TGCGGGAGGCTTTTTGG**TATCTT**
10530	10501	**TTGACT**TCGGTTGAGGGCTTTTGT**TATATT**
13799	13772	**TTGAAA**GAAAACTTTTACTGAT**TATACT**
15793	15765	**TTGACA**GAAAAATCCTCTTAGTC**TATGAT**
25026	24998	**TTGCAT**TCGTGCAGGGGCTTTGT**TATATT**
27762	27735	**TTGACA**CAGCAGCGGAAACCGT**TGTAAA**
31202	31175	**TTGACT**TCATTTCGTAAGTTAC**TACAAT**
31237	31265	**TTGATC**TGATCAAATTTATGATC**TATAAT**
31310	31282	**TTGACC**TACAATGATCATTGTAG**TAAAAT**
31398	31425	**TTGATT**TTGTCTACAGTTATTA**TATAAT**
37508	37535	**TTGACT**TATTAATCAAATGAAC**TATAAT**
47047	47074	**TTGACA**TATAATGTGGGTATGT**TAAAGT**
51361	51389	**TTGACT**CCCTTCTCACATTCCCT**TATAGT**
55495	55523	**TTGCAT**ACGTGCGAGGGCTTTGT**TATATT**
55842	55870	**TTGCTT**ACTGGGTAGTGTAGTGA**TACATT**
71157	71129	**TTGCAT**AGGCTAACGAAGAATAG**TATAGT**
75299	75271	**TTGCGT**TCTCGTGAGGGCTTTTG**TATTAT**
76619	76592	**TTGACA**TCTAAAACGAGAATAC**TATAAT**
77624	77597	**TTGACA**GCGTGAAATGTGATGA**TAGAAT**
78413	78387	**TTGACT**TTATAGCAAAAATAT**TATAAC**
82015	81987	**TTGACT**TCGGTCGGGGGCTTTTT**TATTAT**

82726	82698	**TTGCAT**AAATATAAAGTGGGGTG**TATTAT**
86338	86310	**TTGACA**TCCAGAAAATAAACTAG**TAGTAT**
90583	90555	**TTGCGT**TGTGTGTAGGGTAATGA**TACCTT**
91242	91209	**TTGCTA**AAACTCGATAACCCTGTTCTGT**TATATT**
91279	91252	**TGGAAA**ATCGCTAAGAAAATGG**TATAAT**
91598	91570	**TTGCCA**TTGGCAAGGGGTTTTGT**TATATT**
93293	93265	**TTGCAT**TCGTGCAAGGGCTTTGT**TATATT**
97704	97676	**TTGCAT**TCGTGCAGGGGCTTTGT**TATATT**
100516	100544	**TTGACC**TTTTAAAATCTTTGATC**TATAAT**
100632	100660	**TTGAAT**TCATAAATTTTATGATC**TATAAT**
135204	135176	**TAGTCA**AATTTAAATTGATCTAA**TATAAT**
135198	135225	**TTGACT**ACTAATTAATATAGTA**TATTAT**
138530	138558	**TTGACC**TTACCCTGCTGTTCGAG**TATATT**
141458	141432	**TTGACA**TTAATGATTAAACAT**TATAGT**
142327	142356	**TTGACA**TGCTTTCTGACAGTGGTG**TATGGT**
153971	154000	**TTGACT**TCGGTTGAGGGCTTTTGT**TATATT**
171449	171420	**TTGACT**TCGGTCAGGGGCTTTTGC**TATATT**
173098	173071	**TTGACA**TTCACAAATATGCAAT**TAAAAT**
**Rho-independent terminators of KP27**		
Location		Sequence
Start	End	
3131	3111	GCCTCTTGCAATGCAGGGGGC
6389	6378	CCCCGAAAGGGG
8494	8483	GGGCTTCGGCCC
9509	9529	TCACGGTGTCGCCCACCGTGA
9783	9763	GCCTCTTGCATTGCGGGAGGC
10533	10514	CCCTTGACTTCGGTTGAGGG
17907	17920	GCCCCGAAAGGGGC
17920	17907	GCCCCTTTCGGGGC

20356	20375	TATGGGGCCGAAGCCCCATA
21603	21590	GGGGCTTCGGCCCC
25030	25009	GCCCTTGCATTCGTGCAGGGGC
27015	27002	GGGGCTACGGCCCC
28019	28038	GTGGGGGCCGAAGCCCCCGC
32533	32516	TAGCCCCTTTCGGGGCTA
35763	35784	GCCCATGTAACGAAACGTGGGC
37536	37557	GGGAACTGTTAACGCAGTTCCC
37557	37536	GGGAACTGCGTTAACAGTTCCC
42100	42113	GCCCCTTCGGGGGC
50062	50079	GGCTATCTTCGGATAGCC
50079	50062	GGCTATCCGAAGATAGCC
51313	51334	GCCGTTGCCGAAAGGTAGCGGC
55491	55512	GCCCTTGCATACGTGCGAGGGC
62415	62428	GGGGCTACGGCCCC
62428	62415	GGGGCCGTAGCCCC
64136	64157	GCCCCTTCCTTTCGGTTGGGGC
64157	64136	GCCCCAACCGAAAGGAAGGGGC
70804	70791	GCCCCTTTTGGGGC
75303	75282	GCCCTTGCGTTCTCGTGAGGGC
75342	75371	CCGCACATAAAAGATCATCTATATGTGCGG
78388	78372	CGAGGCTTCGGCCTCG
82019	81989	GCCCTTGACTTCGGTCGGGGGCTTTTTTATT
83529	83514	GGGGGCGAAAGCCCCC
85854	85831	TACCCTGTGTACCCAAACAGGGTA
86690	86677	GCCCCGAAAGGGGC
91602	91582	CCCCTTGCCATTGGCAAGGGG
91582	91602	CCCCTTGCCAATGGCAAGGGG
93297	93276	GCCCTTGCATTCGTGCAAGGGC

93276	93297	GCCCTTGCACGAATGCAAGGGC
97708	97687	GCCCTTGCATTCGTGCAGGGGC
98917	98895	GGGGCTTCGGCCCCTTTTTTATT
101602	101623	GCCCCTTCCTTCCGGTTGGGGC
101623	101602	GCCCCAACCGGAAGGAAGGGGC
109375	109394	CCCCTGTCTTCGGATAGGGG
120050	120063	GGGGCTTCGGCCCC
127561	127576	GGGGTAGCAATACCCC
132922	132939	GGGAACCTTCGGGTTCCC
134974	134999	GGTGGAGTAGGGAAACTTACTCCATC
136119	136098	GCCCCAACCGAAAGGAAGGGGC
136098	136119	GCCCCTTCCTTTCGGTTGGGGC
138928	138941	GGGGCTACGGCCCC
140916	140934	GGGAACCGCAATGGTTCCC
140934	140916	GGGAACCATTGCGGTTCCC
153967	153988	GCCCTTGACTTCGGTTGAGGGC
157684	157707	GGGTGCTCCGGCACCCTTTTTTAT
158940	158953	TGGCCTTCGGGCCA
162939	162922	GGCGGTGTCCCTACCGCC
169308	169326	GGCTACCATATAGGTAGCC
169326	169308	GGCTACCTATATGGTAGCC
171453	171432	GCCCTTGACTTCGGTCAGGGGC
**tRNA of KP27**		
Location		Sequence
Start	End	
49034	49110	GGCCCCTTAGCTCAATAGGTAGAGCTAATCACTCATAATGGTTAGGTTCCCGGTTCAAGTCACGGGAGGGGCCACCA
49116	49192	GGCAAGGCACTTAGAGAAGCGGGAGGAACAGAATCTGATTCTGTTGGTTGTAGGTGCAAATCCTACCCTTGCCACCA

Table S4 Analysis of possible genes involved in DNA modification according to Iyer et al. 2013.

Modification type	GI:	Gene size (bp)	Protein size (aa)	Enzymes	Bases modified	Origin		Identity to YP_007348875	Identity to YP_007348891	Protein of homologue found in KP27 genome
						Phage	Host			
Replacement of thymine by uracil	215788	720	239	Uracil-DNA glycosylase inhibitor (UGI)	Uracil	*Bacillus* phage PBS2	*Bacillus subtilis*	2%	1%	no
Methylation of adenine, cytosine, guanine or uracil	5354346	780	259	DNA adenine-N6 methyltransferase (Dam)	N6-methyladenine (6mA)	*Enterobacteria* phage T4	*Enterobacteriaceae*	0%	0%	yes
	1620188	1035	344	DNA cytosine-C5 methyltransferase (Dcm)	5-methylcytosine (5mC)	*Paramecium bursaria* Chlorella virus 1 (PBCV-1)	*Paramecium bursaria*	0%	0%	yes
	61697902	10	2			*Clostridium* phage phiCD119	*Clostridium difficile*	2%	0%	no
	472341838	702	233	Uracil-5-methyltransferase	5-methyluracil (5mU)	*Cyanophage* MED4-117	-	4%	0%	no
Modification involving thymidylate (dTMP) synthase (TS) family	29366675	741	246	5-hydroxymethylcytosine synthase, deoxycytidylate (dCMP) hydroxymethyltransferase	5-hydroxymethyl-dCMP (5hm-dCMP)	*Enterobacteria* phage T4	*Enterobacteriaceae*	0%	0%	no
	209871285	1152	383	5-hydroxymethyluracil synthase, deoxyuridylate (dUMP) hydroxymethyltransferase	5-hydroxymethyl-dUMP (5hm-dUMP)	*Bacillus* phage SPO1	*Bacillus subtilis*	0%	1%	no
DNA modifications associated with TET/JBP proteins	399528505	867	288	TET/JBP proteins (dioxygenases, hydroxylases)	5-hydroxymethylcytosine (5hmC), 5-hydroxymethyluracil (5hmU)	*Persicivirga* phage P12024L	*Persicivirga sp. IMCC12024*	4%	0%	no
	354997582	879	292	TET/JBP-associated glycosyltrasferases (GTs) (TAGTs)	Glycosyl-5-hydroxymethylcytosine (5ghmC)	*Mycobacterium* phage Acadian	*Mycobacterium smegmatis*	0%	0%	no
	399528508	351	116		Glycosyl-5-hydroxymethyluracil (5ghmU, base J)	*Persicivirga* phage P12024L	*Persicivirga sp. IMCC12024*	2%	0%	no
	354997584	588	195	Amino sugar deacetylases	Deacetylated amino sugar derivatives	*Mycobacterium* phage Acadian	*Mycobacterium smegmatis*	0%	1%	no
	143318241	681	226	Class-II glutamine amidotransferase protein (GAT-II) of the NTN-hydrolase fold	Adenine, guanine or cytosine with a new carbon-nitrogen group	Marine metagenome	*Marine metagenome*	0%	0%	no

Momylation at the N6 position of adenine	143318244	636	211	GCN5-like acetyltransferase (GNAT) superfamily	N6-(1-acetamido)-adenine, N6-carbamoylmethyladenine (6ncmA)	Marine metagenome	*Marine metagenome*	0%	0%	no
Deazapurine-like DNA base modifications	354997289	432	143	6-pyruvoyl tetrahydropterin synthase	hypermodified deazapurines, e.g. 7-cyano-7-deazaguanine (PreQ0)	*Mycobacterium* phage Ares	*Mycobacterium tuberculosis*	0%	0%	no
	314912621	762	253	GTP cyclohydrolase-I (GCHI)	hypermodified deazapurines, e.g. 7-aminomethyl-7-carbaguanine	*Streptococcus* phage Dp-1	*Streptococcus pneumoniae*	0%	0%	no
	29566194	792	263	GTP cyclohydrolase-I (GCHI), QueC-like PP-loop ATPase, QueD-like 6-pyruvoyl-tetrahydropterin synthase, QueE-like radical S-adenosyl methionine (SAM) superfamily protein, guanine transglycosylase family (TGT)	hypermodified deazapurines, e.g. 7-cyano-7-deazaguanine (PreQ0)	*Mycobacterium* phage Rosebush	*Mycobacterium smegmatis*	0%	0%	no
Biosynthesis of α-glutamylthymine or α-putrescinylthymine	418489628	1914	637	5hmU/T synthase, protein with P-loop kinase domain and α-glutamyl/putrescinyl thymine phosphorylase (aG/PT-pyrophosphorylase) domain 1, protein with Nucleotide-modification associated domain 1 (Nmad1)	α-glutamylthymine (α-gluT, agT)	*Bacillus* phage SP10	*Bacillus subtilis*	0%	2%	no
DNA base modification with PP-loop domain	194303201	570	189	IbrA-like pyrophosphate-binding (PP)-loop ATPase	Pyrimidines comparable with lysidine	*Mycobacterium* phage Phaedrus	*Mycobacterium smegmatis*	0%	0%	no
	296389461	576	191	IbrA-like pyrophosphate-binding (PP)-loop ATPase, GCN5-like acetyltransferase (GNAT) superfamily		*Pseudomonas* phage PAb1	*Pseudomonas aeruginosa PAb1*	0%	0%	no
DNA base modification involving ABC ATPase domain	399528443	1140	379	Protein with a N-terminal ABC superfamily ATPase domain and a C-terminal GNAT superfamily domain	Not known	*Persicivirga* phage P12024S	*Persicivirga sp. IMCC12024*	2%	0%	no

	4104915 37	639	212	ABC ATPase, GCN5-like acetyltransferase (GNAT)	Not known	*Salmonella* phage SSU5	*Salmonella spp.*	0%	0%	no
	1342888 13	1131	376	Protein with an N-terminal ABC superfamily ATPase domain and a C-terminal GNAT superfamily domain, formyltransferase	Not known	*Burkholderia* phage φE255	*Burkholderia thailandensis E255*	0%	0%	no
Use of S-adenosylmethionine (SAM) derivatives in synthesis of modified bases	2621137 32	1329	442	S-adenosylmethionine (SAM) synthetase, DNA adenine-N6 methyltransferase (Dam), DNA cytosine-C5 methyltransferase (Dcm), protein with Nucleotide-modification associated domain 4 (Nmad4)	SAM derivatives, e.g. S-adenosyl ethionine	*Streptococcus* phage φ-m46.1	*Streptococcus pyogenes*	5%	1%	no
	3995286 99	1359	452	S-adenosylmethionine (SAM) decarboxylase, DNA adenine-N6 methyltransferase (Dam)	SAM derivatives, e.g. S-adenosyl methioninamine	*Croceibacter* phage P2559S	*Croceibacter atlanticus HTCC2559(T)*	2%	0%	no
DNA base modification involving PBMD1 domain	2655345 0	1095	364	Protein with Predicted base modifying domain 1 (PBMD1)	Not known	*Streptococcus* phage MM1	*Streptococcus pneumoniae*	2%	0%	no
DNA base modification involving aG/PT-pyrophosphorylase biosynthesis	1494082 84	972	323	5hmU/T synthase, pyridoxal 5'-Phosphate Dependent (PLPDE) superfamily enzyme, protein with radical-SAM domain, α-glutamyl/putrescinyl thymine phosphorylase (aG/PT-pyrophosphorylase) 1 and 2, P-loop kinase, protein with Nucleotide-modification associated domain 5 (Nmad5)	Not known	*Pseudomonas* phage M6	*Pseudomonas aeruginosa*	0%	0%	no
	3397556 73	975	324	TET/JBP protein, α-glutamyl/putrescinyl thymine phosphorylase (aG/PT-pyrophosphorylase) 2, P-loop kinase	Not known	*Mycobacterium* phage Zemanar	*Mycobacterium smegmatis*	0%	0%	no
	1894270 11	1047	348	5hmU/T synthase, α-glutamyl/putrescinyl thymine phosphorylase (aG/PT-pyrophosphorylase) 1, glycosyltransferases (GT), sulfotransferase (SulfoT), protein with Nucleotide-modification associated domain 1 (Nmad1)	Not known	*Ralstonia* phage RSL1	*Ralstonia solanacearum*	0%	0%	no

	440789279	1215	404	P-loop kinase, α-glutamyl/putrescinyl thymine phosphorylase (aG/PT-pyrophosphorylase) 2	Not known	*Serratia* phage φMAM1	*Serratia sp.*	0%	0%	no
DNA base modification involving MazG domain	448260061	867	288	α-glutamyl/putrescinyl thymine phosphorylase (aG/PT-pyrophosphorylase), protein with MazG domain (MazG superfamily pyrophosphatase)	Not known	*Dickeya* phage Limestone	*Dickeya solani*	0%	0%	no

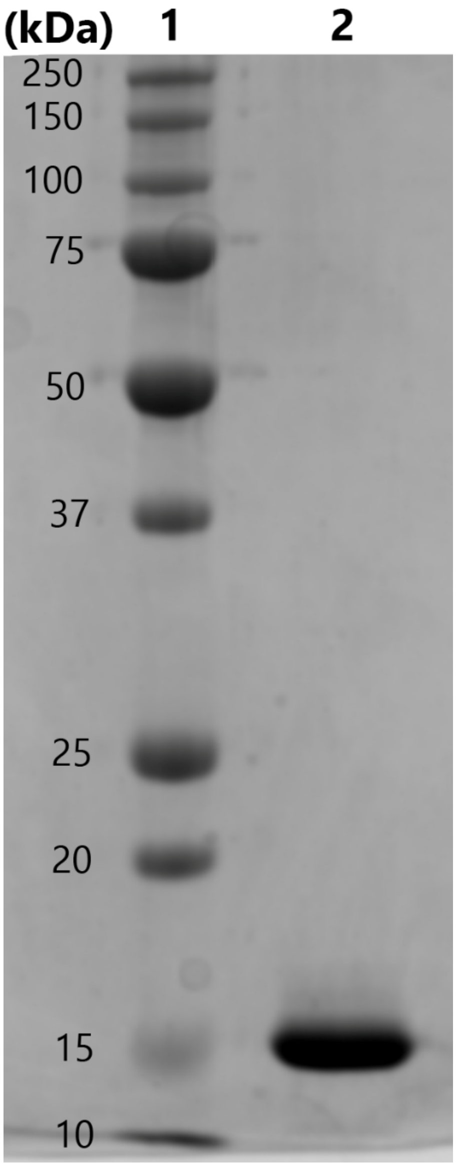

Fig. S1 SDS-PAGE of purified recombinant KP27 endolysin. Mass marker (lane 1); purified KP27 endolysin (lane 2).

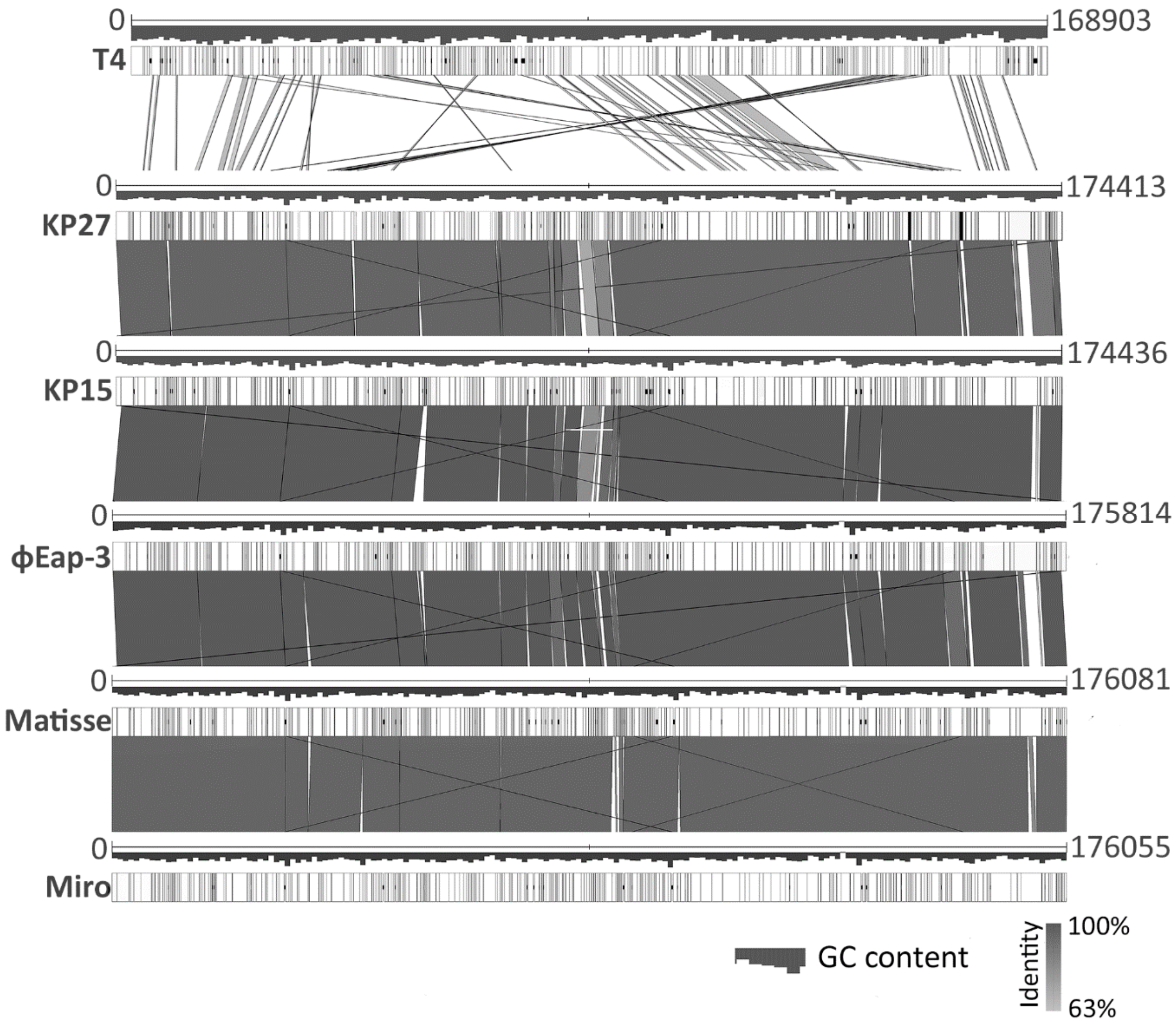

Fig. S2 The genome comparison of “Kp15 virus” representatives.

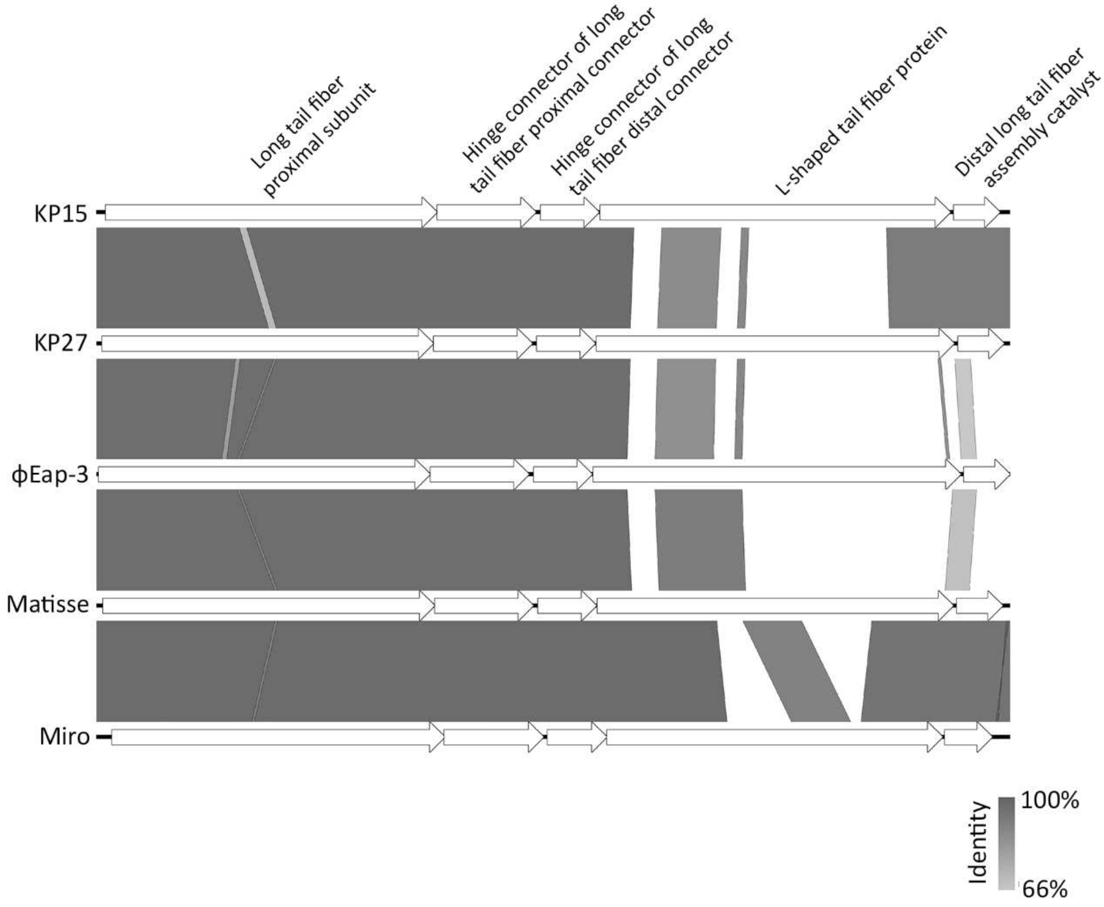

Fig. S3 The similarity of the tail fibers among the “Kp15virus” representatives.

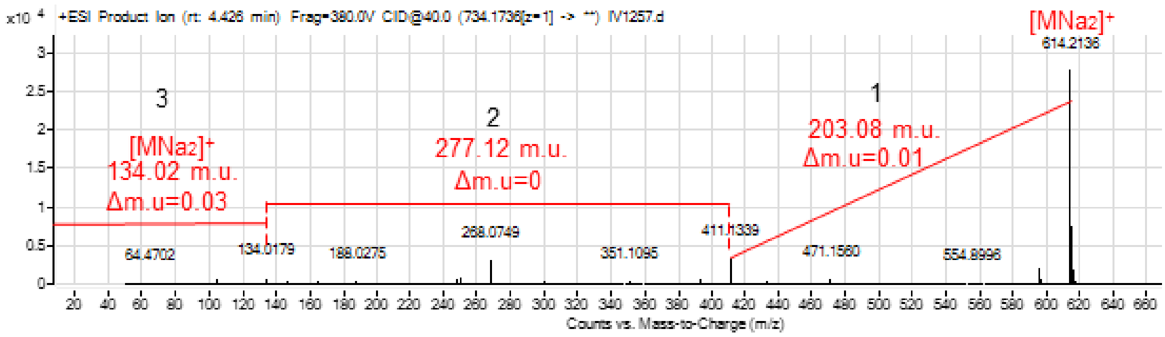

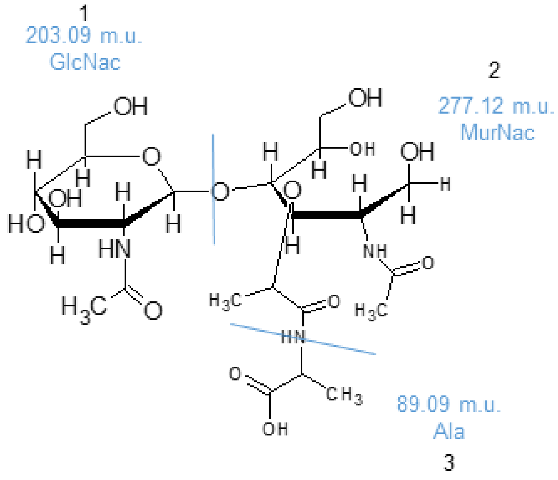

Fig. S4 MS/MS analysis of peak *. It is shown the fragmentation pattern (and the theoretical mass) and a scheme of the structure for the muropeptide. Δm.u.: difference between the experimental (red) and the theoretical mass (blue) value for each fragment.
